# Metagenomic analysis of viromes in honey bee colonies (*Apis mellifera*; Hymenoptera: Apidae) after mass disappearance in Korea

**DOI:** 10.3389/fcimb.2023.1124596

**Published:** 2023-01-25

**Authors:** Minhyeok Kwon, Chuleui Jung, Eui-Joon Kil

**Affiliations:** ^1^ Department of Plant Medicals, Andong National University, Andong, Republic of Korea; ^2^ Agriculture Science and Technology Research Institute, Andong National University, Andong, Republic of Korea

**Keywords:** colony collapse disorder, high-throughput sequencing, honey bee viruses, virome analysis, honey bee (*Apis mellifera* L.)

## Abstract

After the nationwide, massive winter losses of honey bees in Korea during the winter of 2021, samplings were conducted from live honey bees in colonies and dead honey bees nearby colonies in the same bee-farms in six regions in Korea. Each sample was subjected to virome analysis using high-throughput sequencing technology. The number of viral reads was the lowest in the live honey bee group sample with 370,503 reads and the highest in the dead honey bee group sample with 42,659,622 reads. Viral contigs were matched with the viral genomes of the black queen cell virus, deformed wing virus, Israeli acute paralysis virus, and sacbrood virus, all of which have been previously reported in Korea. However, Apis rhabdovirus 5, bee macula-like virus, Varroa orthomyxovirus-1, Hubei partiti-like virus 34, Lake Sinai virus 2, 3, and 4, and the Ditton virus, were also discovered in this study, which are the first records in Korea. Plant viral sequences resembling those of Arabidopsis latent virus 1, and a novel viral sequence was also discovered. In the present study 55 complete viral genome sequences were identified. This study is the first virome analysis of domestic honey bees and provides the latest information on the diversity of honey bee viruses in Korea.

## 1 Introduction

Honey bees are the social insects with distinctive caste and labor divided among queen, workers, and drone bees. Under current beekeeping conditions, tens of thousands of honey bees live together in a narrow cavity nest or hive; consequently, honey bees are highly susceptible to diseases (Tucker, 1978). Of the 100 top food crops that account for 90% of the world’s food, 71% are pollinator-dependent. It has also been estimated that 36% of global crop production are dependent on insect pollinators (Khalifa et al., 2021).

Sacbrood virus was the first virus reported infecting honey bee, *Apis mellifera* in 1913 (White, 1913). Since then, discoveries of honey bee viruses have continued, but they did not receive much attention until colony collapse disorder (CCD) occurred across the United States between winter 2006 and spring 2007 (Oldroyd, 2007). This led to increased research on honey bee viruses (Beaurepaire et al., 2020). The number of honey bee viruses identified before 2007 was 17 (Chen and Siede, 2007), but recently 36 honey bee viruses were introduced in a magazine published in 2021 (Gajda et al., 2021).

Advances in high-throughput sequencing (HTS) technology have increased access to virome analysis and played a major role in the discovery of viruses. HTS technology has opened new horizons in genetic analysis by considerable reducing the extensive cost and time of the previously used Sanger sequencing technology (Pallen et al., 2010; Reuter et al., 2015). Even if the amount of virus replication in the sample is small or infected with a new virus or asymptomatic virus, infected viruses can be detected by virome analysis based on large amount of data using HTS technology (Liu et al., 2011).

Research on honey bee viruses using HTS has been actively conducted; as a result, many viral mutations and novel viruses have been discovered. Remnant et al. (2017) discovered new viruses such as Apis rhabdovirus 1 and 2 through HTS technology. In addition, Mordecai et al. (2016) discovered deformed wing virus (DWV) type C, a new strain of DWV that causes the greatest damage to honey bees, using Illumina sequencing, an HTS technique. Gauthier et al. (2015) discovered the entire genome of dsDNA replicated in honey bees using the Illumina system from a dsDNA virus (Apis mellifera filamentous virus). Other researchers are now also searching for honey bee viruses using HTS technology (Levin et al., 2016; Remnant et al., 2017; Roberts et al., 2018; Thaduri et al., 2018; Ryabov et al., 2019; Gusachenko et al., 2020; Kadlečková et al., 2022; Lester et al., 2022).

In Korea, research on honey bee viruses has focused only on the surveillance of disease incidences utilizing the reported virus sequence information for last decade. Significant alerts on the genetic diversity and local temporal variation were initiated with the sacbrood virus epidemics in *Apis cerana* from 2009 in Korea (Yoo and Yoon, 2009; Choe et al., 2012a; Choe et al., 2012b; Ouh et al., 2013; Reddy et al., 2013; Kim et al., 2016). Sequences of novel honey bee viruses and new isolates/strains of previously known viruses in *Apis mellifera* have been reported in neighboring countries, including China and Japan, but rarely conducted in Korea yet (Zhang et al., 2001; Morimoto et al., 2012; Kitamura and Asai, 2022). In 2021 winter, nationwide massive winter losses of honey bees occurred with numerous projections of the causal inferences (Jung and Bae, 2022; Kim, 2022; Lee et al., 2022). Just after the winter, we collected honey bee samples from live honey bee in colonies and dead honey bee nearby colonies as well in the damage inflicted bee farms, and investigated virome using HTS technology to identify the occurrence of new and unrecorded viruses.

## 2 Materials and methods

### 2.1 Honey bee sample collection and RNA extraction

Honey bee samples were collected between March 31 and April 3, 2022, from Yeongwol in Gangwon-do, Yeongdong in Chungcheongbuk-do, Gunwi in Gyeongsangbuk-do, Geochang in Gyeongsangnam-do, and Hwasun and Gangjin in Jeollanam-do (**Figure S1**). At one apiary per region, live honey bees in the hives that suffered CCD were randomly sampled at ten times by designating group “A” and dead honey bees in the vicinity as group “B” (no sample corresponding to group “B” in Gunwi). The collected honey bees were placed in 15 ml conical tubes containing 70% alcohol and stored at 4°C. Three honey bees from each sample were randomly ground using a mortar, and RNA was extracted using the RNA extraction protocol of TRIzol^®^ (Thermo Fisher Scientific, Massachusetts, US) proposed by Toni et al. (2018). The extracted RNA was stored in a ULT Freezer 900 (Thermo Fisher Scientific) at -80°C until analysis, after checking the concentration and purity with a NanoPhotometer^®^ NP80 (IMPLEN, California, US).

### 2.2 High-throughput sequencing (HTS)

Before HTS, quality control (QC) was ensured using a 2100 Bioanalyzer (Agilent, California, US), and libraries were constructed using TruSeq Stranded Total RNA with Ribo-Zero H/M/R Gold (Illumina, California, US). After the libraries were created libraries, their quality was checked using the 2100 Bioanalyzer. Samples that passed QC were analyzed using an Illumina NovaSeq 6000 (Illumina).

### 2.3 Virome analysis

Raw reads obtained through HTS technology were analyzed using the CLC Genomics workbench software (version. 22.0.1, QIAGEN, Hilden, Germany). Unnecessary parts of raw reads of each sample were first trimmed through ‘Trim reads’. After that, the host genome of the honey bee (*Apis mellifera*) was downloaded from the NCBI Genome database (NCBI, 2022b), the host genome was mapped to trimmed reads, and unmapped reads were collected. Then, the virus information registered in the NCBI database (NCBI, 2022a) was downloaded, and unmapped reads were mapped to the virus information. To identify novel viruses that were not registered in the NCBI database, contigs were created through *de novo* assembly using unmapped reads. Contigs were extracted from the data showing E-values from 0 to *xe*-10 from tBLASTx results using the virus database and conducted by the NCBI BLASTn process to find novel viruses. The novel viruses were classified based on species demarcation criteria of the International Committee on Taxonomy of Viruses (ICTV).

### 2.4 Validation of novel virus using RT-PCR

Based on the viral sequences found in the virome analysis, primer sets were designed using the NCBI Primer-BLAST tool (https://www.ncbi.nlm.nih.gov/tools/primer-blast/; **Table 1**). RT-PCR was carried out using SuPrimeScript RT-PCR Premix (2×) (Genetbio, Daejeon, Korea) with 7 µL of RNase-free water, 1 µL of forward primer, 1 µL of reverse primer, and 1 µL of template RNA for 40 cycles, and the annealing temperature was used differently for each primer (**Table 1**). PCR products were electrophoresed on 1% agarose gel in Mupid^®^-2plus (ADVANCE, Tokyo, Japan) at 90 V under 100 VAC for 25 min, and a 100 bp DNA ladder (Bioneer, Daejeon, Korea) was used as the size marker. After electrophoresis, the gel was read using GelDoc Go (Bio-Rad, California, US).

**Table 1:** List of primers used in this study.

Virus	Sequence	Annealing temp (°C)	Size (bp)
BQCV	F 5’-AAATGCCAATGTGGACCAAA-3’	55	370
	R 5’-GATAGGGCTGCTATCCACCG-3’		
DWV	F 5’-CGGTGCGACTGAAACTTCTA-3’	55	610
	R 5’-CATACGTTCTTGCTCCAGCG-3’		
IAPV	F 5’-ATTCCTGTGTCGGAGCAGTG-3’	55	870
	R 5’-CAAAGTATCCTCAAGTTGTGGG-3’		
SBV	F 5’-GGAGGCCTGGGAAAAGAGTG-3’	55	535
	R 5’-TTCCAACTGCACCACAGGTT-3’		
VOV-1 PB1	F 5’-AGTAGTGGTCTCCCAGTAGG-3’	55	920
	R 5’-CTGTGTACCGATTTTGCACCC-3’		
VOV-1 PB2	F 5’-AAGAGAAGCATGTGGCCCAA-3’	55	516
	R 5’-GGAGCTCACGGACTTTGTCA-3’		
VOV-1 PA	F 5’-GTACAAGCACCTGCATTCGTG-3’	55	514
	R 5’-GGCCTGTAGAGAGGACCAGA-3’		
VOV-1 glycoprotein	F 5’-AAACCAACCGGTTCTTGGTG-3’	54	526
	R 5’-TGGCACTTTGCTGGTTTATAATG-3’		
VOV-1 nucleoprotein	F 5’-AAAGGAGCCGAGCATTGTCA-3’	55	631
	R 5’-CCACGTCTAGCCCCTTCAAT-3’		
VOV-1 M protein	F 5’-ACCAGAGACAGCCACCTTTC-3’	55	246
	R 5’-AGGATGCACTCCCTTCAGGT-3’		
LSV2	F 5’-TTTGCTCTAGTCGTGTGCGT-3’	55	505
	R 5’-CCAGCCGACCGTAAGGATAC-3’		
LSV3	F 5’-ATCTGCTCCCTGGAACTGGG-3’	56	246
	R 5’-GAGGGTGAGGAGATGGGACT-3’		
LSV4	F 5’-CCCATGGGTGGTGAGATCGA-3’	56	690
	R 5’-CGACGACGAGAACGGGAGGT-3’		
ARV5	F 5’-TGGAGCTGTAAGAGAGGATGTG-3’	55	400
	R 5’-TCGGCATGACTAGAGGATCAC-3’		
BeeMLV	F 5’-TCCCCTTCCTTCTCAAAGCA-3’	55	892
	R 5’-CGCCTGAAAGGTGGATGAGT-3’		
HPLV34	F 5’-TGCGTGTGTAAAAATCACTGGA-3’	55	457
	R 5’-CGGTGGAAGTGTGGAGAGAC-3’		
AmPLV1	F 5’-AACAAGCACTGGAGGAGACG-3’	55	882
	R 5’-AGCATCCGTGTTCATTCGGT-3’		
DV	F 5’-ATGTCAACTGGTGGCTCGAC-3’	55	638
	R 5’-AGACTTTGCATATCCTCCCTTTACA-3’		
AmCV RNA1	F 5’-CAATGCGCCACATGGACTTG-3’	55	856
	R 5’-TGAAACAAGTCCCGCCATCA-3’		
AmCV RNA2	F 5’-CAAGTGCGCTATAACACGCC-3’	55	642
	R 5’-GACGCACTCCAAAAACCACC-3’		

### 2.5 Phylogenetic analysis and pairwise comparison

A phylogenetic tree was constructed using the viral sequence information in MEGA 11 software (version 11.0.13) and the complete sequence information of each virus was registered in the NCBI GenBank. Phylogenetic trees were created using the maximum likelihood method (Murshudov et al., 1997), and 1000 bootstrap iterations were applied for nucleotide distance measurement (Jukes and Cantor, 1969). In the case of novel viruses, a phylogenetic tree was constructed by referring to the sequences of viruses registered in the ICTV for comparison with viruses belonging to each viral genus.

### 2.6 TCS network

TCS analysis (Clement et al., 2002) was performed using Population Analysis with Reticulate Trees (PopART, version 1.7) (Leigh and Bryant, 2015). The nexus file for TCS analysis was extracted using CLC Genomics Workbench software, and each group was divided by checking the phylogenetic tree and pairwise comparison table of each virus to create the traits file.

## 3 Results

### 3.1 Identified virus-derived reads from all samples

**Figure 1:**
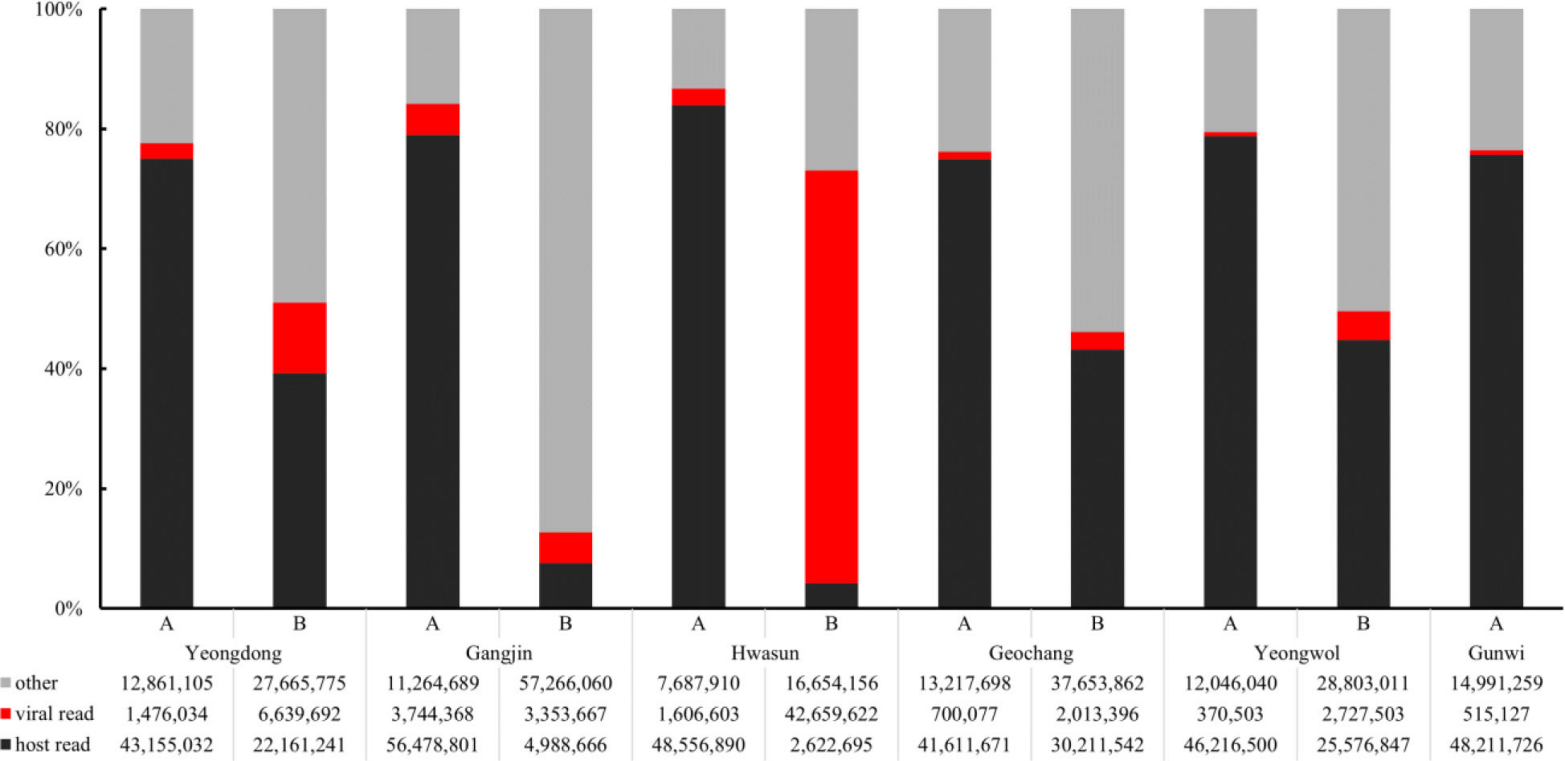
Comparison of read counts from six regions. Numbers of host reads (in black), viral reads (in red), other reads (in gray) were distinguished. Group “A” has fewer viral reads than Group “B”.

From the virome analysis, the average number of reads in the sample after trimming was 61,428,161. In the case of viral reads, the average of group A was 1,402,119, and the largest read number among group A was identified in Gangjin (3,744,368). The average read number of group B was 11,478,776, and the sample from Hwasun had the highest number of reads (42,659,622; **Figure 1**). As a result of analyzing which virus the read was derived from among the viruses previously reported in Korea, black queen cell virus (BQCV, one sample, 9%), deformed wing virus (DWV, four samples, 36%), Israeli acute paralysis virus (IAPV, five samples, 45%), and sacbrood virus (SBV, one sample, 9%) were identified (**Table 2**). Among previously unreported viruses in Korea, Apis rhabdovirus 5 (ARV5, two samples, 18%), bee macula-like virus (BeeMLV, one sample, 9%), Hubei partiti-like virus 34 (HPLV34, two samples, 18%), Varroa orthomyxovirus-1 (VOV-1, three samples, 27%), Lake Sinai virus 2 (LSV2, two samples, 18%), Lake Sinai virus 3 (LSV3, eight samples, 72%), Lake Sinai virus 4 (LSV4, five samples, 45%) and Ditton virus (DV, one sample, 9%) were found, and Apis mellifera associated partiti-like virus 1 (AmPLV1, one sample, 9%) and Apis mellifera associated comovirus (AmCV, two samples, 18%) were also identified as novel viruses (**Table 2**). In conclusion, virome analysis identified 55 complete cds sequences in 11 samples, and 26 sequences were found in group A, including two novel virus sequences. In group B, 29 sequences were found, including three novel virus sequences (**Table S1**).

**Table 2:** Numbers of mapped reads matched with viral genomes.

Collection site		Reported virus in Korea				Unreported virus in Korea									Plant virus
		BQCV	DWV	IAPV	SBV	ARV5	VOV-1	BeeMLV	HPLV34	LSV2	LSV3	LSV4	AmPLV1	DV	AmCV
**Yeongdong**	A	–	–	–	–	–	49,112	–	–	802,460	–	–	–	–	891
	B	2,401	613,959	1,437	4,043,993	9,628	–	–	–	–	1,447,775	49,298	119	–	4,770
**Gangjin**	A	–	–	–	–	–	–	–	–	267,526	1,464,714	1,848,997	–	–	–
	B	–	1,527	–	–	–	–	–	–	–	3,044	–	–	–	–
**Hwasun**	A	–	–	1,056,726	–	–	20,435	–	–	–	–	–	–	–	–
	B	–	16,433,499	25,586,868	–	–	16,646	1,239	–	–	17,356	–	–	–	–
**Geochang**	A	–	1,604	623	–	–	–	–	–	–	–	–	–	–	–
	B	–	–	–	–	–	–	–	–	–	463,370	3,166	–	13,799	–
**Yeongwol**	A	–	–	–	–	–	–	–	144,715	–	1,050	1,674	–	–	–
	B	–	–	–	–	13,405	–	–	162	–	718,020	528,021	–	–	–
**Gunwi**	A	–	–	11,474	–	–	–	–	–	–	225,124	–	–	–	–
**Total**	A	–	1,604	1,068,823	–	–	69,547	–	144,715	1,069,986	1,690,888	1,850,671	–	–	891
	B	2,401	17,048,985	25,588,305	4,043,993	23,033	16,646	1,239	162	–	2,649,565	580,485	119	13,799	4,770

### 3.2 Identification of a novel virus (*Partitiviridae*)

A contig found in Yeongdong of group B was 1,325 nt in length and showed 96% similarity to the uncultured virus (JF732915) belonging to the *Partitiviridae* family and 70% similarity to Vespa velutina associated partiti-like virus 2 (MN565048) as a result of BLASTn. A tBLASTx of the RdRp region showed 71.99% identity with Vespa velutina associated partiti-like virus 2 (MN565048; **Table S2**). Based on the similarity result with the existing sequence, the partitivirus identified in Yeongdong of group B was named as a new species ‘*Apis mellifera associated partiti-like virus 1* (AmPLV1, OP972921)’.

### 3.3 Revalidation of HTS results by RT-PCR

To confirm the analysis of the virome results from HTS, RT-PCR was performed using primers designed based on the viral genome sequences identified in this study. As a result of performing RT-PCR on viruses found in samples from each region, bands at the target size were confirmed in all samples, except for samples in which some reads were read low (DWV from Gangjin of group B and BeeMLV from Hwasun of group B; **Figure S2**).

### 3.4 Identification of plant-infecting comovirus

From tBLASTx, four contigs showed the highest similarity with Arabidopsis latent virus 1 (ArLV1; MH899120 and MH899121), belonging to the subfamily *Comovirinae*, family *Secoviridae*. Contigs from Yeongdong of group A showed 76.28% and 75.71% similarity to RNA1 and RNA2, respectively, whereas contigs from Yeongdong of group B showed 76.50% and 75.85% similarity to RNA1 and RNA2, respectively (**Table S3**). The lengths of each segment were confirmed to be 5,899 nt (RNA1, OP972917) and 3,442 nt (RNA2, OP972919) for Yeongdong of group A, and 5,921 nt (RNA1, OP972918) and 3,608 nt (RNA2, OP972920) for Yeongdong of group B (**Figure S3**). Compared with other viruses belonging to the subfamily *Comovirinae*, it was confirmed that the comovirus-related contigs in this study were closely related to ArLV1 (**Figures S4**, **S5**). These comovirus-related sequences were judged to be novel, and were given a new name, ‘*Apis mellifera associated comovirus* (AmCV)’.

### 3.5 Reported honey bee viruses in Korea

Regarding the four viruses that have been previously reported in Korea (BQCV, DWV, IAPV, and SBV), phylogenetic trees and pairwise comparison tables were constructed using CLC Genomics Workbench with each complete viral sequence reported to NCBI GenBank by October 2022 (**Figures S6**, **S7**, **S8**, **S9**, **S10**, **S11**, **S12**, **S13**). The complete genome sequence of BQCV identified in Yeongdong of group B (OP972872) was confirmed to have the closest affinity to the sequences reported in China among previously reported sequences (**Figure S6**), and showed the highest similarity to the Tianjin isolate (MZ821816, 97.43%; **Figure S7**). For DWV, the viral contigs showed the closest relationship with isolates previously reported in Korea (**Figure S8**). Complete genome sequences from Yeongdong of group B (OP972876, 97.31%), Geochang of group A (OP972874, 97.04%), and Gangjin of group B (OP972873, 94.35%) had the highest similarity to the Korean isolate (JX878305), and the sequence from Hwasun of group B (OP972875) was confirmed to be most similar to the Neimenggu isolate (MZ821833, 97.32%; **Figure S9**). IAPV sequences from Hwasun of group A and B (OP972881, 97.70% and OP972882, 97.20%), Geochang of group A (OP972880, 96.55%), and Gunwi of group A (OP972916) had the closest relationship to the Heilongjiang isolate (MZ821840), and a sequence isolated from Yeongdong of group B (OP972883) was most similar to the Hubei isolate (MZ821841, 98.29%; **Figures S10**, **S11**). The SBV sequence identified in Yeongdong of group B (OP972898) was similar to those reported in isolates from Australia (KY465671, KY465675, KY465676, KY465677, and KY465678), Jeonju (JQ390591), and Ulsan (KP296800; **Figure S12**). Among them, the South Australian isolate (KY465677, 97.31%) showed the highest similarity to the SBV sequence from Yeongdong of group B (**Figure S13**).

### 3.6 Unreported honey bee viruses in Korea

Similarity comparisons were also conducted between the unreported seven virus species (ARV5, BeeMLV, HPLV34, VOV-1, LSV2, 3, and 4) and isolates reported abroad. ARV5 has been reported only in China thus far, and the sequences of Yeongdong and Yeongwol of group B (OP972869 and OP972870) confirmed in this study were 99.77% similar compared to each other and 97%-98% similarity with the isolate reported in Chinese (MZ822106-8; **Figures S14**, **S15**). The BeeMLV sequence identified in Hwasun of group B (OP972871) showed a 90.94% similarity to the China Jilin isolate (MZ821799), but showed a low similarity with the French (NC_027631) and USA isolates (KT162925) of 75.26% and 65.45%, respectively (**Figures S14**, **S15**). For HPLV34, only the Italian (MT747982) and Chinese (KX884207) isolates have been reported to date. The sequences identified in Yeongwol of group A and B (OP972878 and OP972879) were 94.27% and 96.22% similar to the Italian isolate, respectively, and the Chinese isolate showed 96.56% and 98.28% similarity, respectively (**Figures S14**, **S15**). In the case of VOV-1, six segments were found in Yeongdong of group A, Hwasun of group A, and B, and each segment was compared using a phylogenetic tree and a pairwise comparison table. The VOV-1 segment PA gene sequence identified in Yeongdong of group A (OP972904, 99.06%) was similar to the China Shanxi isolate (MZ822037); Hwasun of group A (OP972902, 98.72%) was similar to the China Hebei isolate (MZ822007), and B (OP972903, 99.36%) showed the same similarity to the China Shanxi and Hebei isolates (**Figures S16**, **S17**). The VOV-1 segment PB1 gene sequence identified in Yeongdong of group A (OP972907, 95.01%), Hwasun of group A (OP972905, 96.80%), and B (OP972906 97.42%) was similar to the China Shanxi isolate (MZ822036) and a 70% similarity to the Israeli isolate (MK032466; **Figures S16**, **S17**). However, the VOV-1 segment PB2 gene sequence identified in Yeongdong of group A (OP972910, 95.66%), Hwasun of group A (OP972908, 95.70%), and B (OP972909, 98.17%) was similar to the China Shanxi isolate (MZ822035) and showed 85%-87% similarity with that of the Czech Republic isolate (OL803854; **Figures S16**, **S17**). The VOV-1 segment glycoprotein gene sequence identified in Yeongdong of group A (OP972913, 90.26%), Hwasun of group A (OP972911, 96.10%), and B (OP972912, 95.57%) was similar to the China Hebei isolate (MZ822008) and showed 41% similarity was confirmed with the Israeli isolate (MK032468; **Figures S16**, **S17**). The VOV-1 segment nucleoprotein gene sequence identified in Yeongdong of group A (OP972923, 97.47%), Hwasun of group A (OP972914, 97.34%), and B (OP972915, 97.80%) was similar to the China Shanxi (MZ882039) and Czech Republic (OL803852) isolates, and the three samples showed ​​86%-87% similarity (**Figures S16**, **S17**). The VOV-1 segment M protein sequence identified in Yeongdong of group A (OP972900, 96.83%), Hwasun of group A (OP972899, 98.69%), and B (OP97290198.29%) was similar to the China Shanxi (MZ822040) and the Czech Republic isolates (OL803855; 69%-70%, **Figures S16**, **S17**).

Three Lake Sinai viruses (LSV2, 3, and 4) were also analyzed and compared to foreign isolates. For LSV2, Yeongdong of group A (OP972884) had the highest similarity with the China isolate (MT732482, 98.02%) and Gangjin of group A (OP972922) had the highest similarity with the China Shanxi isolate (MZ821853, 86.95%) but both the Korean isolates showed 85.80% similarity when compared with each other (**Figures S18**, **S19**). For LSV3, Yeongdong of group B (OP972890, 94.91%) showed high similarity to the China Shandong isolate (MZ821866), identified in Yeongwol of group B (OP972892, 98.81%) and Gangjin of group B (OP972886, 97.31%) showed similarity to the China Hunan isolate (MZ821863). Gangjin of group A (OP972885, 97.11%), Hwasun of group B (OP972889, 96.91%), Geochang of group B (OP972894, 97.57%), Gunwi of group A (OP972887, 96.60%), and Yeongwol of group A (OP972891, 95.98%) showed similarity to the China Hebei isolate (MZ821854; **Figures S20**, **S21**). LSV4 from Yeongdong of group B (OP972895) and Gangjin of group A (OP972893) showed high similarity to the China Liaoning isolate (MZ821905, 95.85% and 98.07%, respectively). Yeongwol of group A (OP972896) was similar to the China Fujian isolate (MZ821871, 97.63%); Geochang of group B (OP972894) was similar to the China Tianjin isolate (MZ821852, 96.39%), and Yeongwol of group B (OP972897) was similar to the China Jilin isolate (MZ821893, 93.29%; **Figures S22**, **S23**). A contig found in Geochang of group B (OP972877) was 7,701 nt in length and was similar to the Ditton virus (DV) isolate (MF893264, 98%; **Figure S24**).

### 3.7 TCS haplotype network results

**Figure 2:**
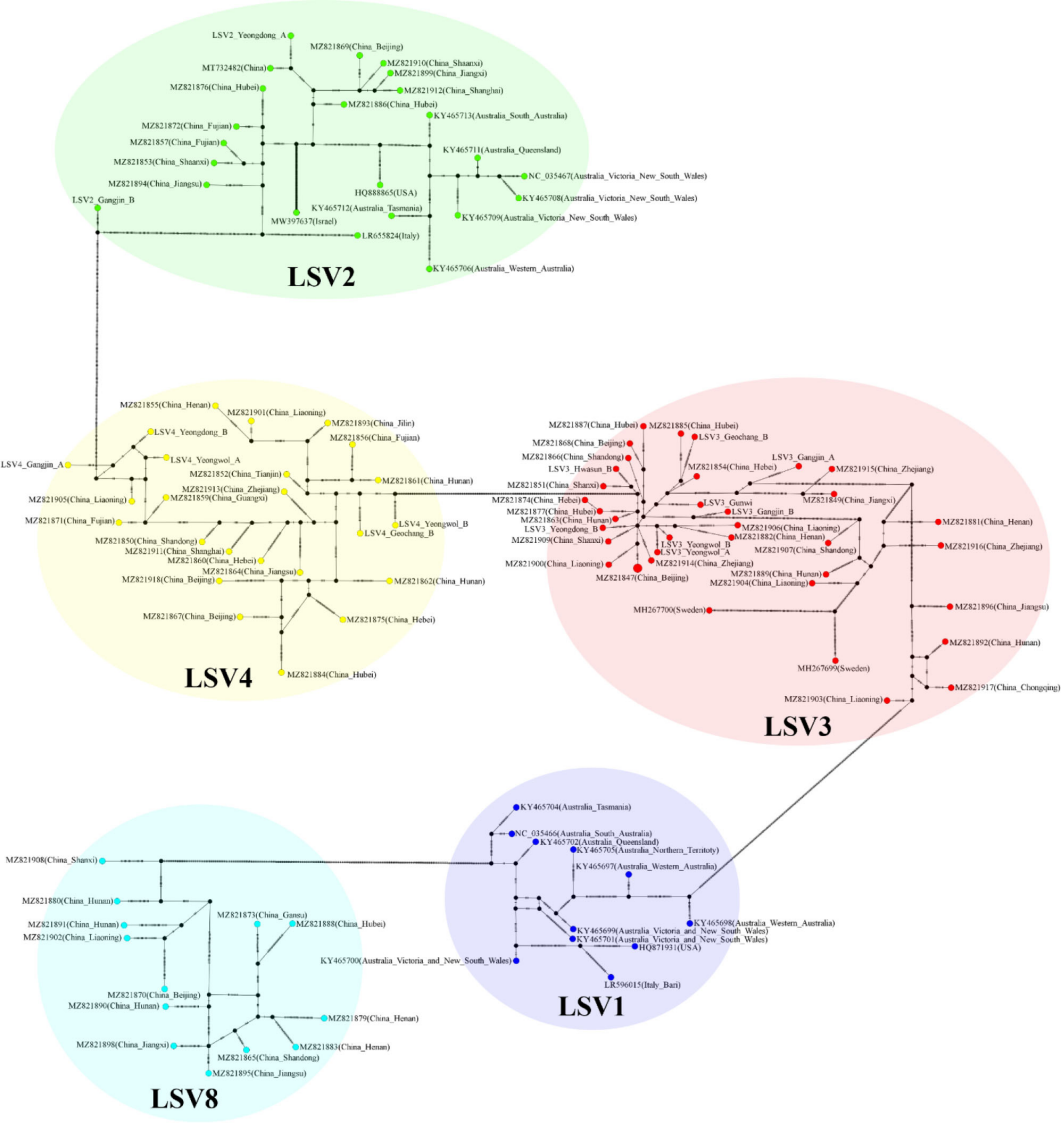
TCS haplotype network, with nodes colored by different Lake Sinai virus 1, 2, 3, 4 and 8 (LSV1, 2, 3, 4, and 8) RNA-dependent RNA polymerase (RdRp) regions clade. A total of 108 sequences for LSV1, 2, 3, 4 and 8 RdRp regions were used to construct the TCS network. The perpendicular dashes on the branches connecting two nodes represent the number of nucleotides difference between those nodes. This TCS network is analyzed and visualized by PopART.

**Figure 3:**
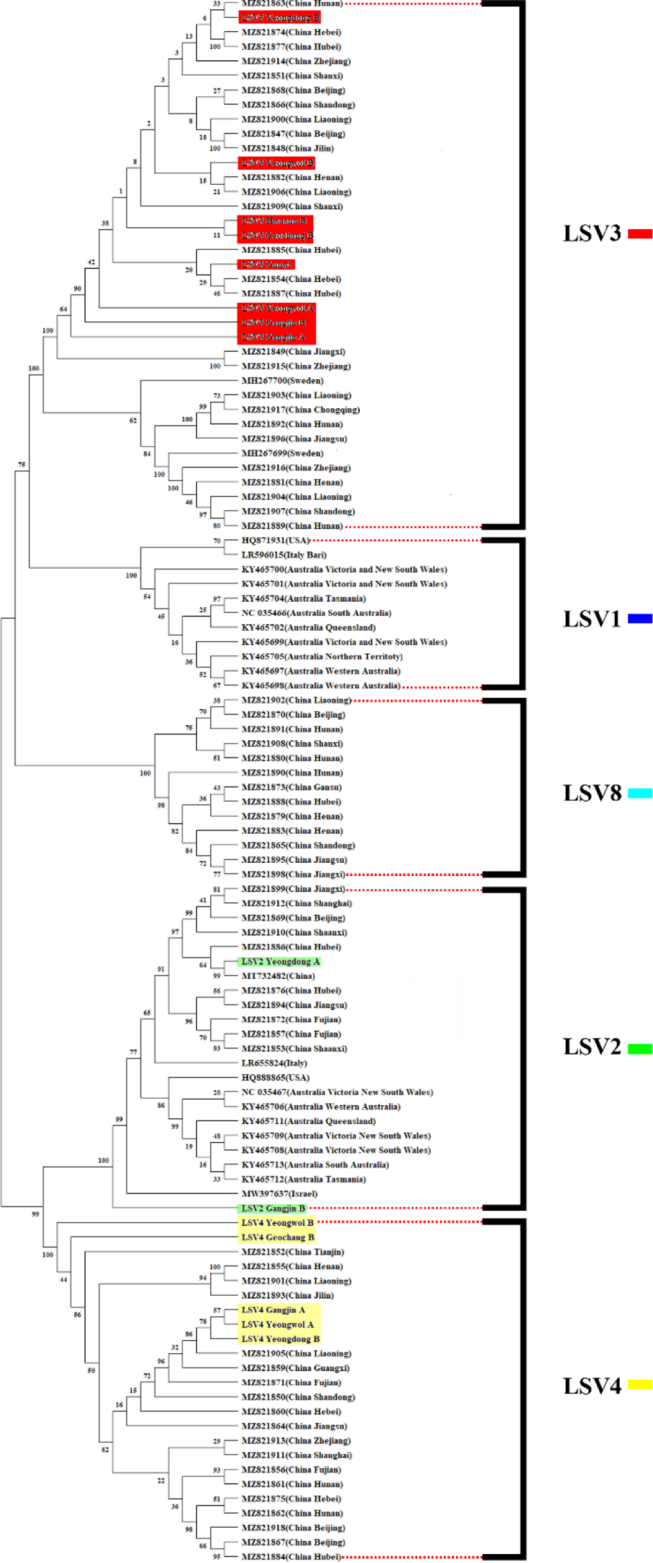
Phylogenetic tree visualizing and describing the relatedness for RNA-dependent RNA polymerase (RdRp) regions genetic relationships between Lake Sinai virus 1, 2, 3, 4 and 8 (LSV1, 2, 3, 4, and 8) sequences using maximum likelihood method. The tree was developed using LSV2 (green), LSV3 (red), LSV4 (yellow) sequences detected in virome analysis, and LSV1, 2, 3, 4 and 8 (black) with complete sequences reported in the NCBI GenBank database. The bootstrap value was from 500 replicates and nucleotide distance was measured by Jukes-Cantor method. This tree is analyzed and visualized by MEGA11 using 94 sequences reported in NCBI GenBank database and 14 sequences found in this study.

**Figure 4:**
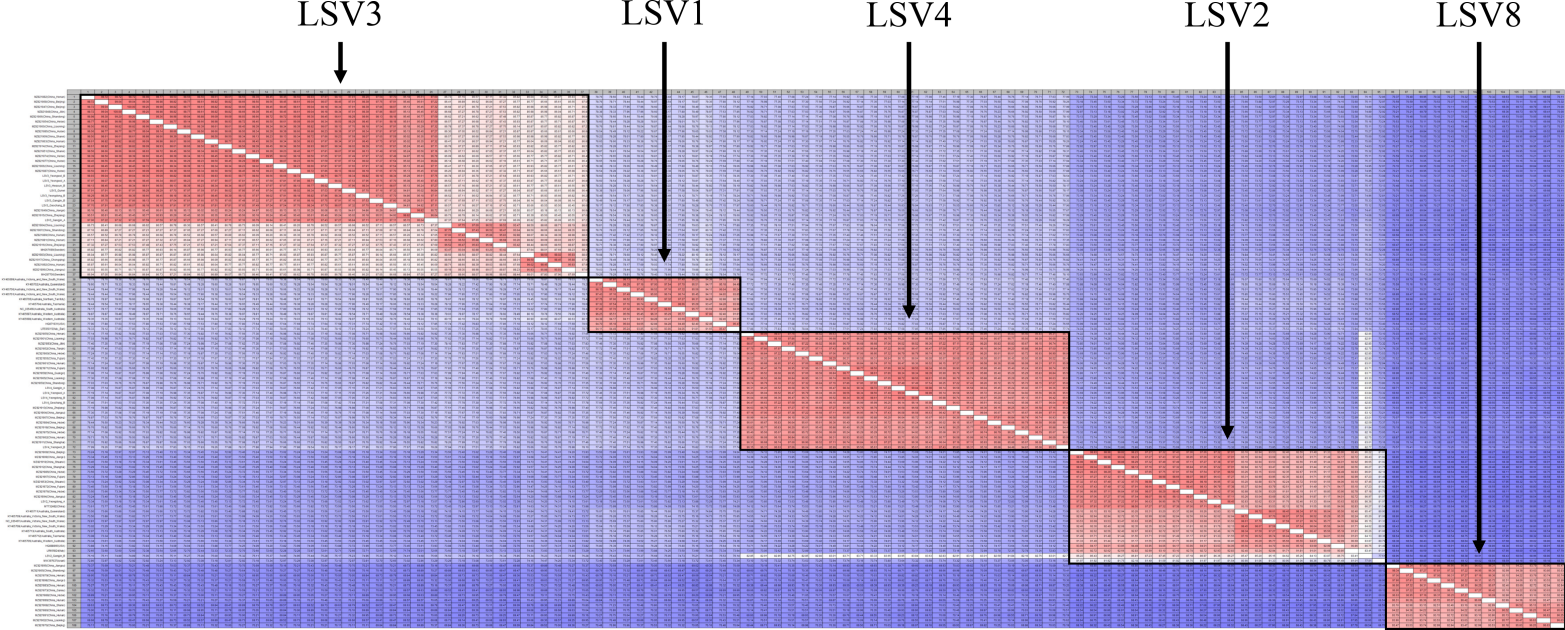
Pairwise comparison table using RNA-dependent RNA polymerase (RdRp) region among Lake Sinai virus 1, 2, 3, 4 and 8 (LSV1, 2, 3, 4, and 8) complete sequences for each country reported to NCBI GenBank database using CLC Genomics Workbench. Percent identity in 11,664 pairwise comparisons among 108 sequences (15 sequences detected in this study and 93 sequences reported from NCBI GenBank database).

The TCS haplotype network and phylogenetic tree were verified by referring to the pairwise comparison table for the LSV group, which confirmed that LSV1, 2, 3, 4, and 8 were associated with each virus (**Figures 2**–**4**). The criteria to distinguish between species within the LSV group are currently unclear, but they can be distinguished based on their similarity to each other. LSV2 was grouped into four clades. Clades 1 and 2 were connected and stretched from clade 4. In clade 4, the nucleotide differences between each virus were confirmed to be lower than that of clade 3 (**Figure S25**). LSV3 was grouped into three clades. It was confirmed that there was a large difference in nucleotides between the clades. Clade 3 included many nodes; however, some viruses showed severe nucleotide variations (**Figure S26**). LSV4 was grouped into two clades. LSV4 found in Yeongwol of group B showed that clade 2 had more nucleotide variations than other viruses (**Figure S27**). BQCV was grouped into three clades. In the four viruses divided into clade 1, the nucleotide sequence variation was higher than that of the other viruses (**Figure S28**). DWV was grouped into three clades. Clades 1 and 2 had large nucleotide variations, and the viral genome sequence reported from the Egyptian sample, where DWV was first discovered, was bound to clade 1 alone (**Figure S29**). IAPV was grouped into two clades. All four viral genome sequences detected in this study were divided into clade 2; however, Yeongdong of group B was further from the other three sequences (**Figure S30**). SBV was grouped into three clades. The viral genome sequence of clade 2 showed significant nucleotide variation. In this study, the viral genome sequences identified in Yeongdong of group B belonged to clade 3 (**Figure S31**).

## 4 Discussion

The health of honey bees is an important topic related to worldwide food production in a rapidly changing climate. Substantial threats from habitat losses, malnutrition, pesticides, pests and diseases and other environmental factors were reported associated with the colony collapse disorder (Botías et al., 2015). Pesticide interactions have emerged as a major threat to the health of honey bees, especially on the neonicotinoid pesticides on seed dressing (Johnson et al., 2010; Pisa et al., 2015). However, the pests and diseases cannot be ignored on this issues, for examples, varroa mite and Israeli acute paralysis virus and so on (Ellis, 2012; Cornelissen et al., 2019).

In Korea, there has been little interest in novel viruses as only PCR-based investigations of seven major viruses have been conducted on honey bees. Therefore, starting with several honey bee viruses discovered in this study, it is necessary to investigate unrecorded viruses and novel viruses in Korea by including more regions in future studies.

In the present study, 55 complete viral genome sequences were identified. Among them, there is a virus believed to be a plant virus. Four major viruses (BQCV, DWV, IAPV, and SBV) have been detected for diagnosis in Korea. Among them, DWV and IAPV should be noted. Researchers have suggested that DWV would highly associated with the winter losses since this is mainly vectored by the parasitic varroa mites and indicating the varroa mite pressure (Highfield et al., 2009; Natsopoulou et al., 2017). It has also been reported that IAPV is directly related to CCD (Cox-Foster et al., 2007). As the massive losses and disappearance of honey bees in Korea has some phenomenon similarity to CCD in the Western world, every possible causal inference should be pursued. DWV and IAPV were the most frequently detected viruses in Korea, and represented the majority of reads compared to other viruses. In addition, both viruses are closely related to CCD (Cox-Foster et al., 2007; Schroeder and Martin, 2012) and are thought to have a close relationship with the previous mass disappearance of honey bees in Korea; therefore, follow-up studies on these viruses are essential. Forty complete viral genome sequences have been detected in honey bees which have not been previously reported in Korea. The symptoms of the discovered viruses have not yet been identified. Additional tests are required to determine the risk posed by these viruses.

DV are first discovered in *Drosophila suzukii*. However, the previously reported DV did not have a complete ORF with a partial sequence, but the contig identified in this study could confirm the complete ORF.

The LSV group (LSV2, 3, and 4) is the most frequently detected virus group in this study and have received considerable attention in recent studies (Šimenc et al., 2020; Shojaei et al., 2021; Čukanová et al., 2022; Kitamura and Asai, 2022). LSV3 was the only virus detected in all the regions in this study. Although the symptoms have not yet been identified, the first LSV discovered was a honey bee sample that had CCD (Runckel et al., 2011). Therefore, recent studies suggest that it is necessary to consider the potential correlation between LSV and CCD (Daughenbaugh et al., 2015; Faurot-Daniels et al., 2020). In Korea, a CCD-like phenomenon occurred in the winter of 2021, and several LSV groups were found in Korea. Therefore, it will be necessary to continuously check the occurrence of LSVs as major diagnostic target viruses.

Viruses identified by HTS based virome analysis were assayed using RT-PCR. However, no bands were identified using RT-PCR for DWV (Gangjin B) and BeeMLV (Hwasun B). It was impracticable to confirm the RT-PCR results because of the small number of reads corresponding to the target virus using virome analysis. In addition, all Gunwi A RNA samples were used for HTS analysis, and it was impossible to test for viruses identified in the analysis.

The honey bees used in this study were collected from the live honey bee in colonies and dead honey bee nearby colonies from only six regions in spring. The sampling amount would not be sufficiently enough to visualize the distribution of honey bee viruses by region or period. In addition, since virus level varies according to the season (Dainat et al., 2012; Steinmann et al., 2015; Desai and Currie, 2016), a study on the change in virus accumulation in seasonal samples is required. However, this study introduces the results of the first honey bee virome analysis conducted in Korea and is considered an important basis for future related studies.

## Data availability statement

The datasets presented in this study can be found in online repositories. The names of the repository/repositories and accession number(s) can be found in the article/**Supplementary Material**.

## Author contributions

E-JK and CJ conceived and designed the study. MK performed the experiments. MK conducted the bioinformatics and data analysis. MK and E-JK wrote the manuscript. All authors contributed to manuscript revision and read and approved the submitted version.
