# Supplementary material for: Metagenomic analysis of viromes in honey bee colonies (Apis mellifera; Hymenoptera: Apidae) after mass disappearance in Korea
Source: Front Cell Infect Microbiol. 2023 Jan 25;13:1124596. doi: 10.3389/fcimb.2023.1124596 (PMC9905416; doi:10.3389/fcimb.2023.1124596)
Supplement: Supplementary file 1 [file DataSheet_1.docx]

Supplementary Material

Metagenomic analysis of viromes in honey bee colonies (*Apis mellifera;* Hymenoptera*:* Apidae) after mass disappearance in Korea

Minhyeok Kwon, Chuleui Jung, Eui-Joon Kil^*^

*** Correspondence:** Eui-Joon Kil, viruskil@anu.co.kr


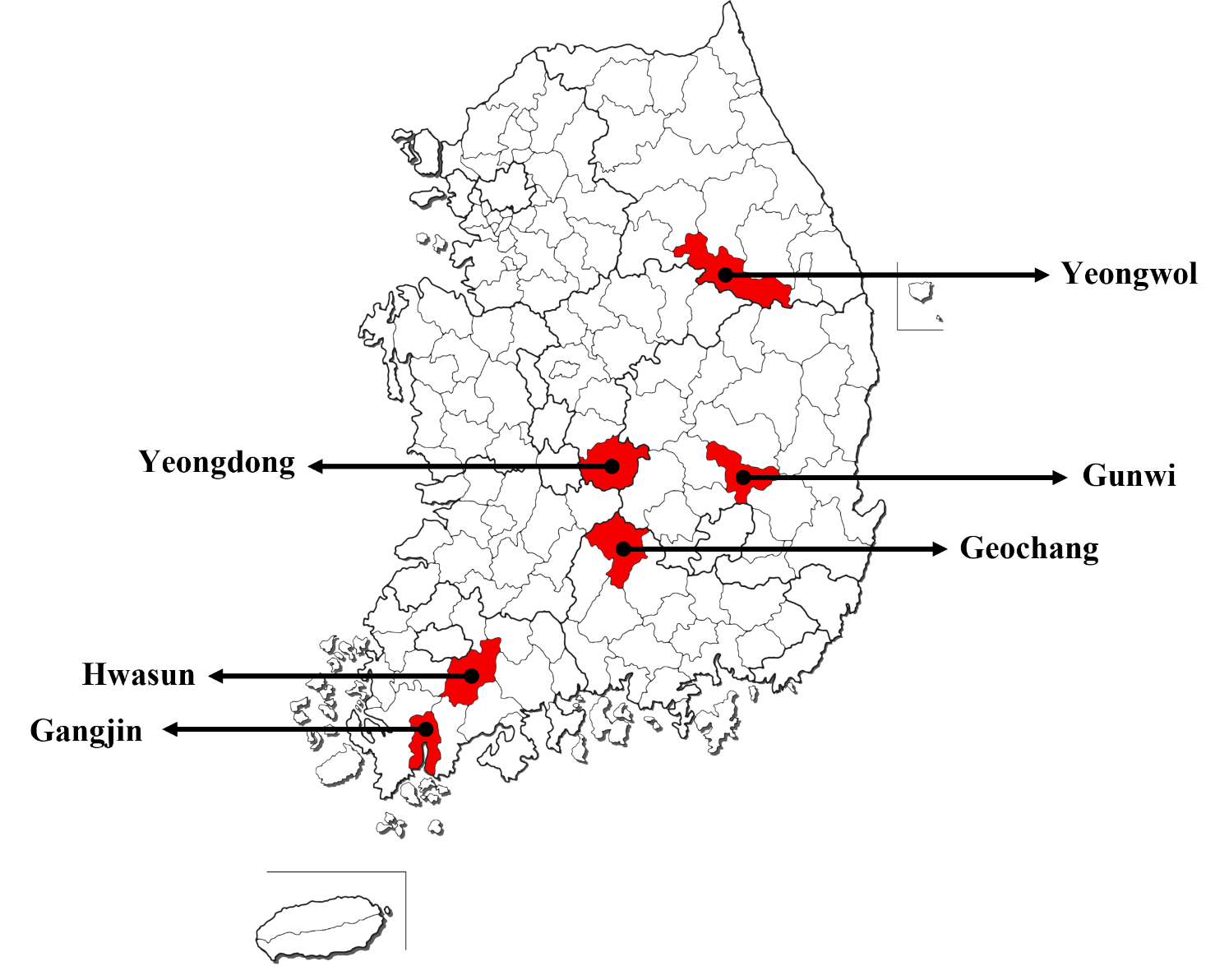


**Fig. S1** Sampling site locations for honey bee virus surveys. It was collected from hives that suffered CCD at one honey bee farm per region. Live honey bee inside as group “A”. Dead honey bee in the vicinity as group “B”.


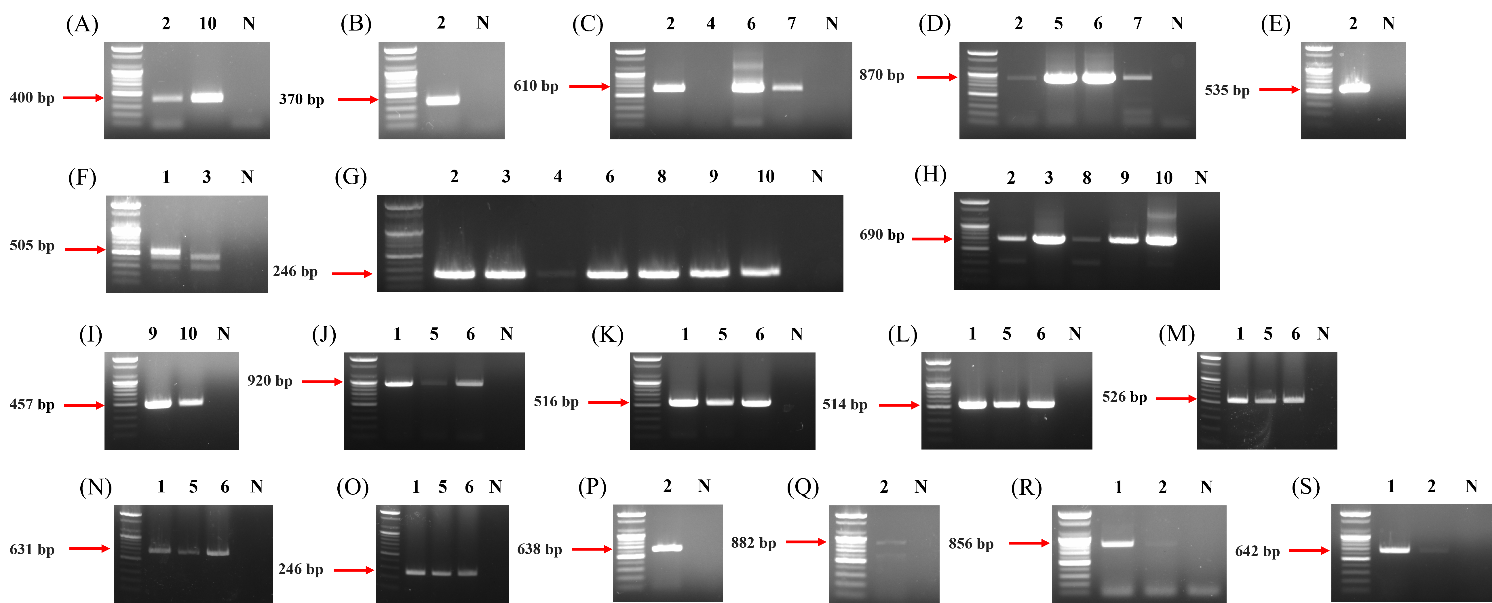


**Fig. S2** Verification results using RT-PCR of the virus found in virome analysis. (A) Apis rhabdovirus 5 (ARV5). (B) Black queen cell virus (BQCV). (C) Deformed wing virus (DWV). (D) Israeli acute paralysis virus (IAPV). (E) Sacbrood virus (SBV). (F) Lake Sinai virus 2 (LSV2). (G) Lake Sinai virus 3 (LSV3). (H) Lake Sinai virus 4 (LSV4). (I) Hubei partiti-like virus 34 (HPLV34). (J) Varroa orthomyxvirus-1 (VOV-1) PA gene. (K) VOV-1 PB1 gene. (L) VOV-1 PB2 gene. (M) VOV-1 glycoprotein gene. (N) VOV-1 nucleoprotein gene. (O) VOV-1 M protein gene. (P) Ditton virus (DV). (Q) Apis mellifera associated partiti-like virus 1 (AmPLV1) (R) Apis mellifera associated *Comovirus* (AmCV) RNA1. (S) Apis mellifera associated *Comovirus* (AmCV) RNA2. Lane 1, Yeongdong of group A; lane 2, Yeongdong of group B; 3, Gangjin of group A; 4, Gangjin of group B; 5, Hwasun of group A; 6, Hwasun of group B; 7, Geochang of group A; 8, Geochang of group B; 9, Yeongwol of group A; and 10, Yeongwol of group B.


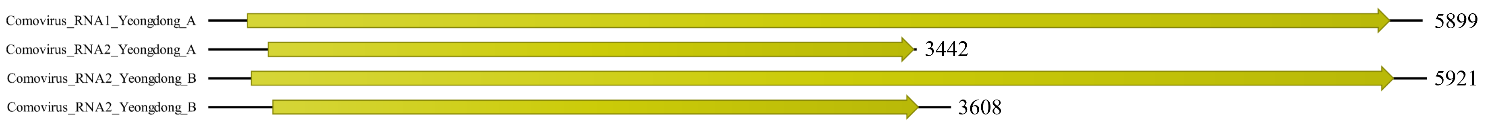


**Fig. S3** Genome structure of viral contigs identified in Yeongdong. RNA1 contains the Pro-Pol coding region. RNA2 contains the CP coding region.


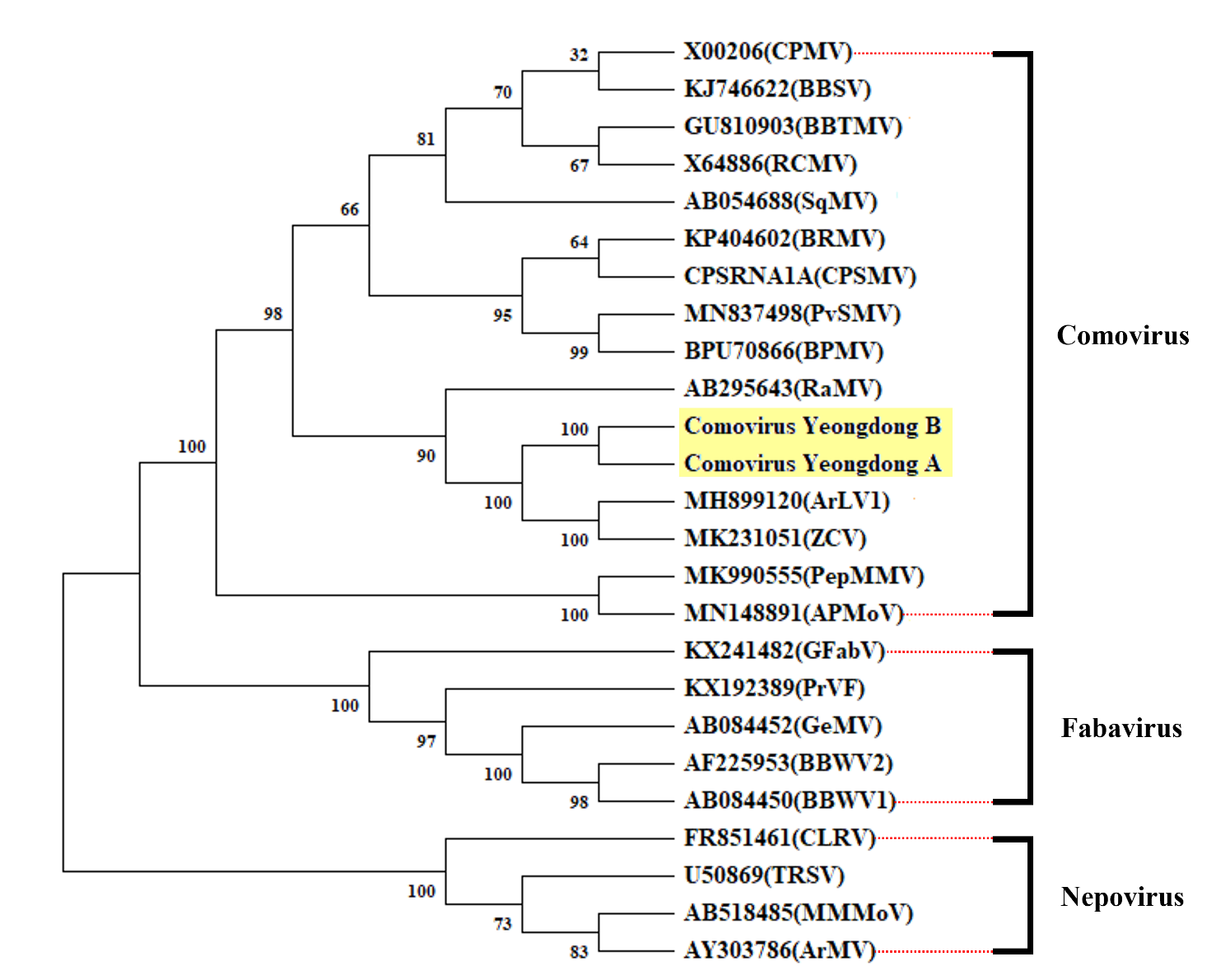


**Fig. S4** Phylogenetic trees illustrating the relations of comovirus-associated contigs with RNA1 of other species in the subfamily *Comovirinae* reported in ICTV. Phylogenetic tree method is neighbor-joining and 1000 times bootstrap, nucleotide distance measure using Jukes-Cantor method. The tree was developed using Apis mellifera associated comovirus (AmCV) sequences (highlighted in yellow) from detected from virome analysis and other reference sequences reported from the NCBI GenBank database using MEGA11. The detected virus was included in the genus *Comovirus* and the most similar to ArLV1 was confirmed. For analysis, 23 sequences reported in the NCBI GenBank database and two sequences found in this study were used.


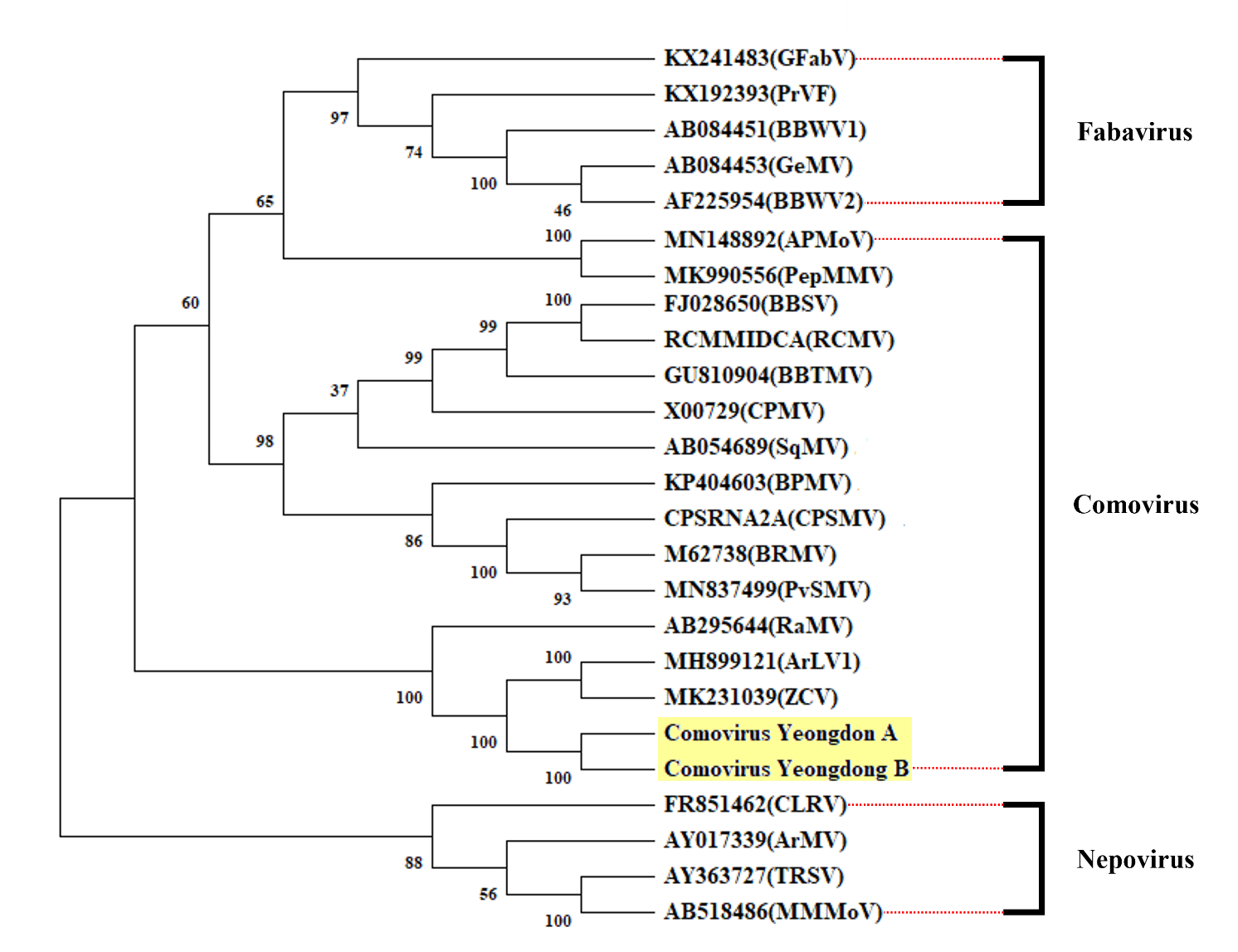


**Fig. S5** Phylogenetic trees illustrating the relations of comovirus-associated contigs with RNA2 of other species in the subfamily *Comovirinae* reported in ICTV. Phylogenetic tree method is maximum likelihood and 1000 times bootstrap, nucleotide distance measure using Jukes-Cantor method. The tree was developed using Apis mellifera associated comovirus (AmCV) sequences (highlighted in yellow) from detected from virome analysis and other reference sequences reported from the NCBI GenBank database using MEGA11. The detected virus was included in genus *Comovirus* and ZCV was most resemble virus. For analysis, 23 sequences reported in the NCBI GenBank database and two sequences found in this study were used.


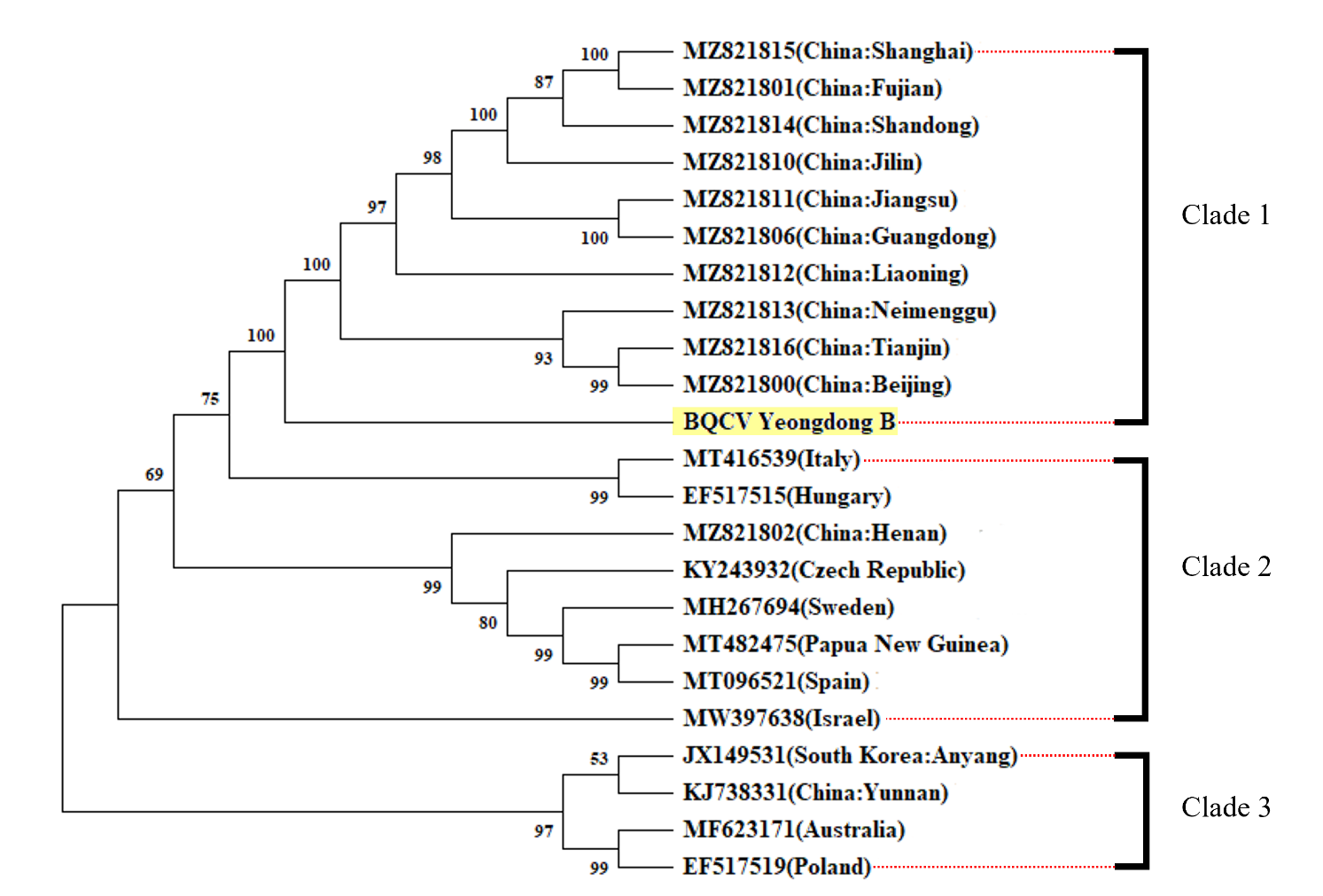


**Fig. S6** Phylogenetic tree visualizing and describing the relatedness for black queen cell virus (BQCV) sequences using maximum likelihood method. The tree was developed using BQCV sequences (highlighted in yellow) detected from virome analysis and other reference sequences reported in the NCBI GenBank database. The bootstrap value was from 1000 replicates and nucleotide distance was measured by Jukes-Cantor method. This tree is analyzed and visualized by MEGA11. For analysis, 22 sequences reported in the NCBI GenBank database and one sequence found in this study were used.


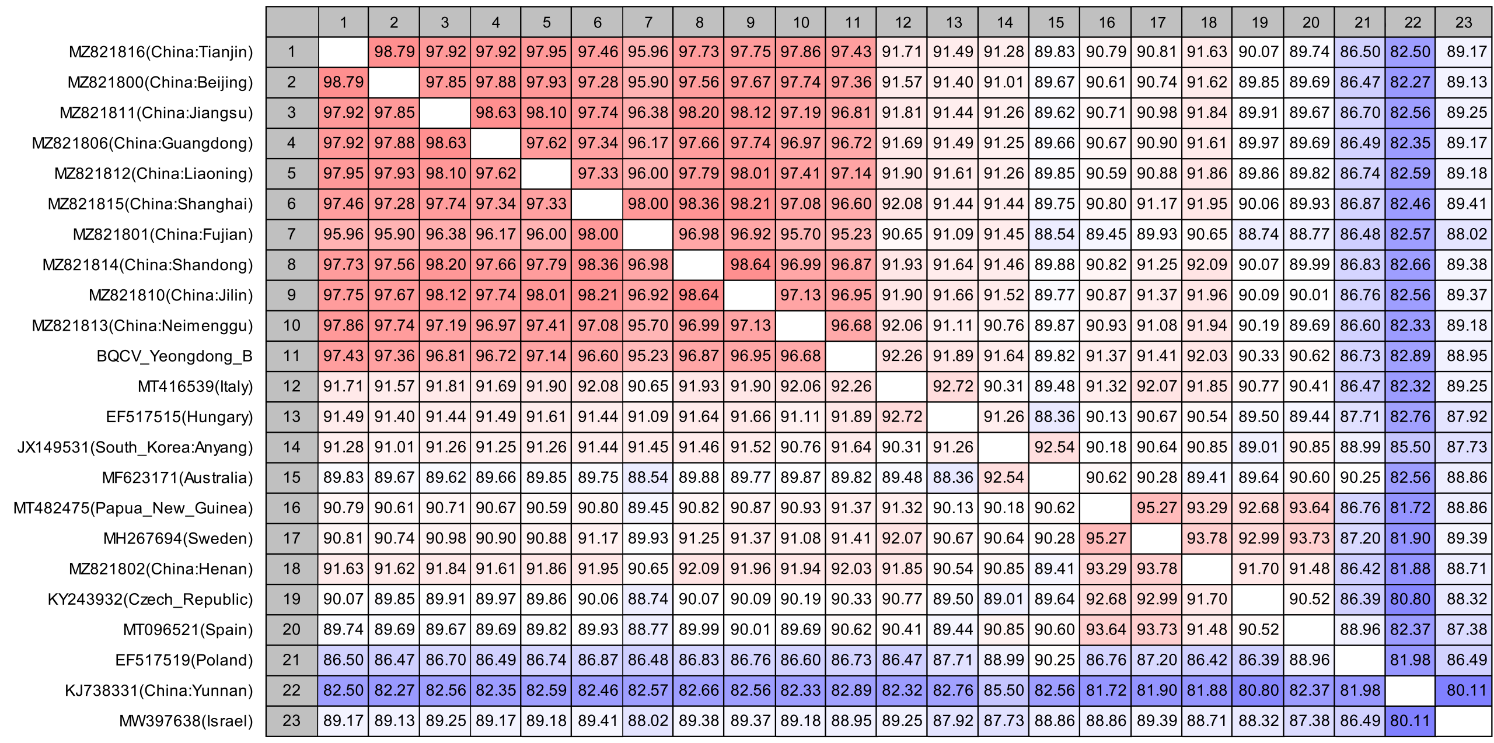


**Fig. S7** Pairwise comparison table using Black queen cell virus (BQCV) complete sequence for each country reported to NCBI GenBank database using CLC Genomics Workbench. Percent identity in 529 pairwise comparisons among 23 BQCV sequences (one sequence detected in this study and 22 sequences reported from NCBI GenBank database).


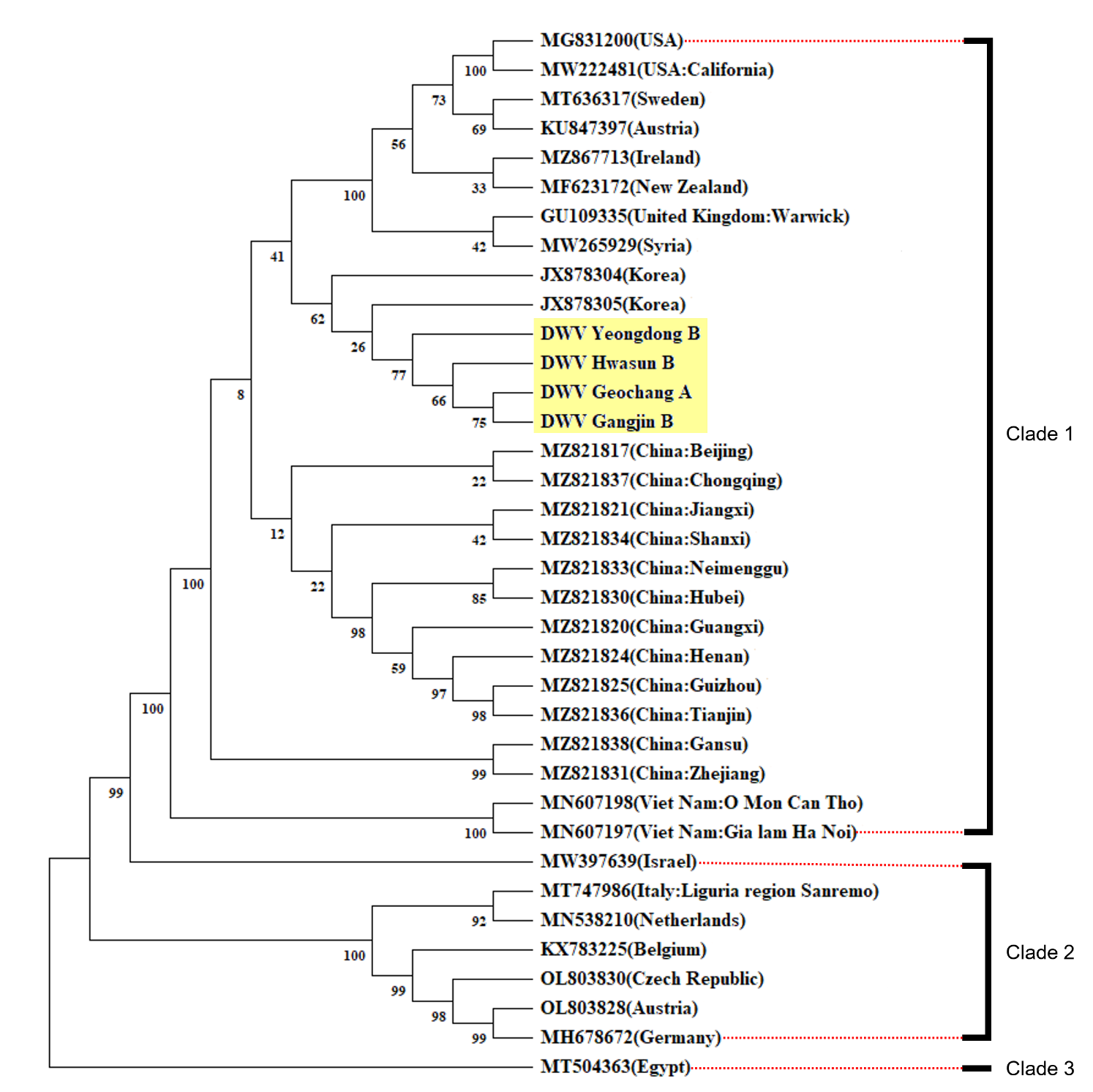


**Fig. S8** Phylogenetic tree visualizing and describing the relatedness for deformed wing virus (DWV) sequences using maximum likelihood method. The tree was developed using DWV sequences (highlighted in yellow) detected from virome analysis and other reference sequences reported in the NCBI GenBank database. The bootstrap value was from 1000 replicates and nucleotide distance was measured by Jukes-Cantor method. This tree is analyzed and visualized by MEGA11. For analysis, 32 sequences reported in the NCBI GenBank database and four sequences found in this study were used.


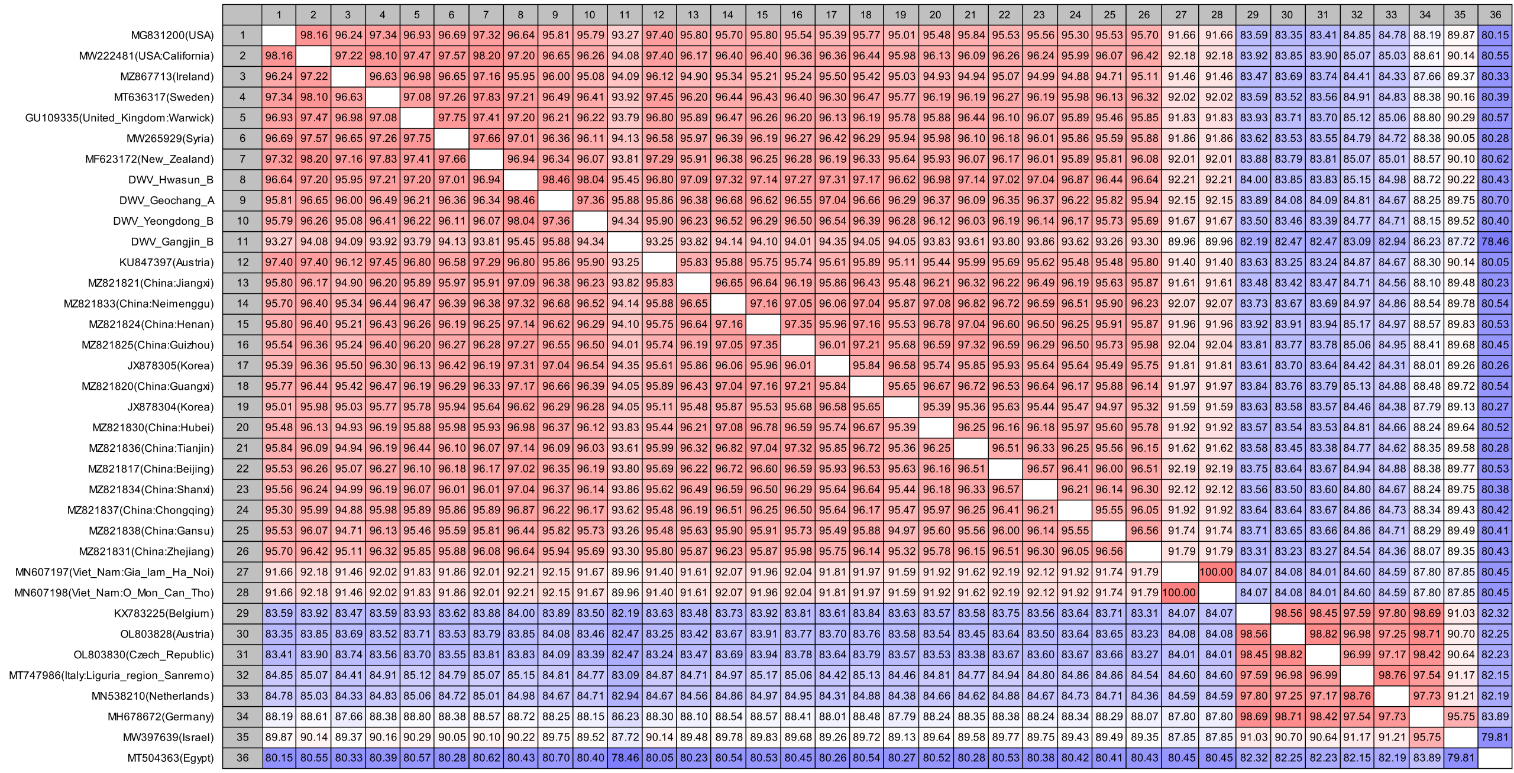


**Fig. S9** Pairwise comparison table using deformed wing virus (DWV) complete sequence for each country reported to NCBI GenBank database using CLC Genomics Workbench. Percent identity in 1,296 pairwise comparisons among 36 DWV sequences (four sequences detected in this study and 32 sequences reported from NCBI GenBank database).


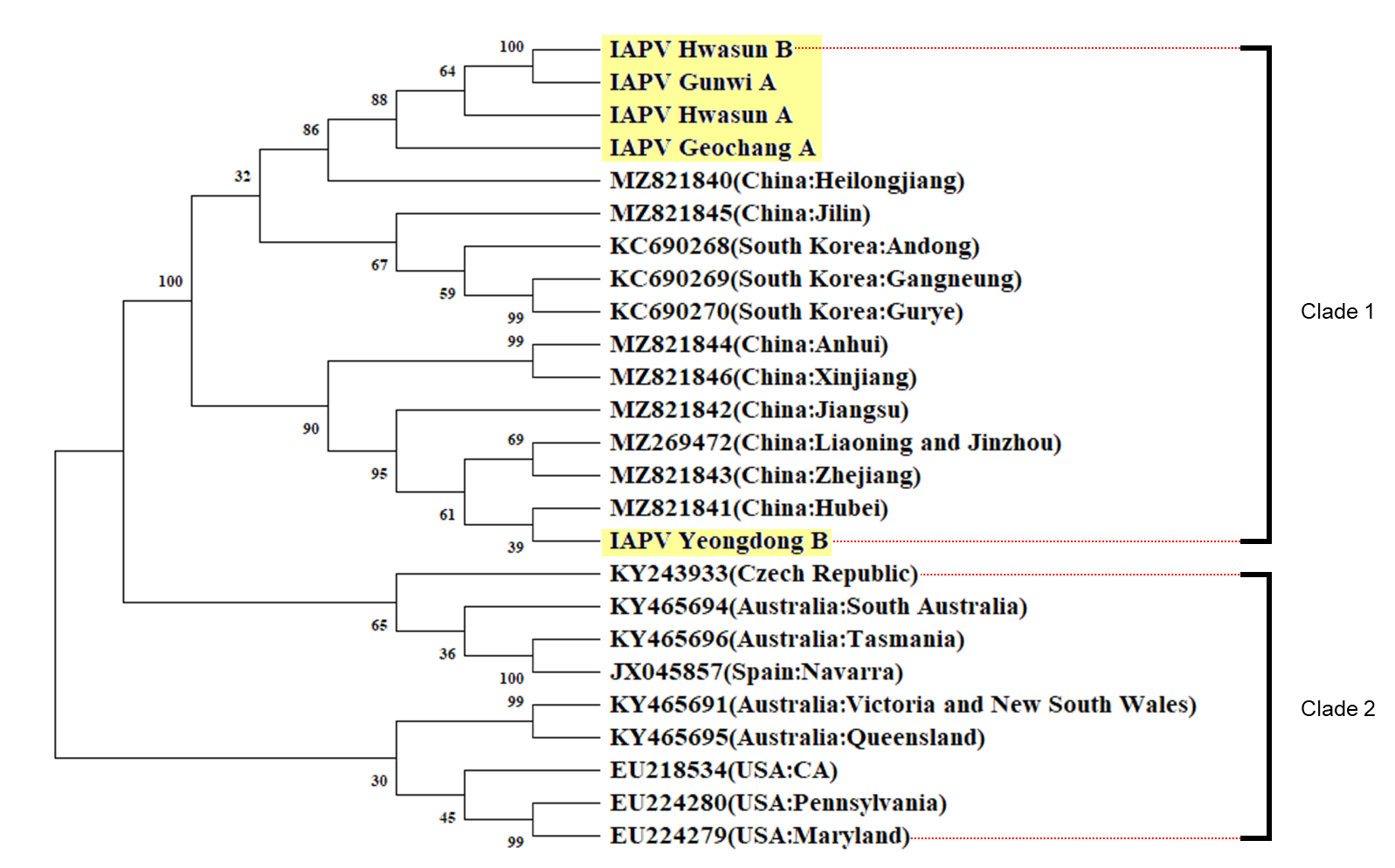


**Fig. S10** Phylogenetic tree visualizing and describing the relatedness for Israeli acute paralysis virus (IAPV) sequences using maximum likelihood method. The tree was developed using IAPV sequences (highlighted in yellow) detected from virome analysis and other reference sequences reported in the NCBI GenBank database. The bootstrap value was from 1000 replicates and nucleotide distance was measured by Jukes-Cantor method. This tree is analyzed and visualized by MEGA11. For analysis, 25 sequences reported in the NCBI GenBank database and five sequences found in this study were used.


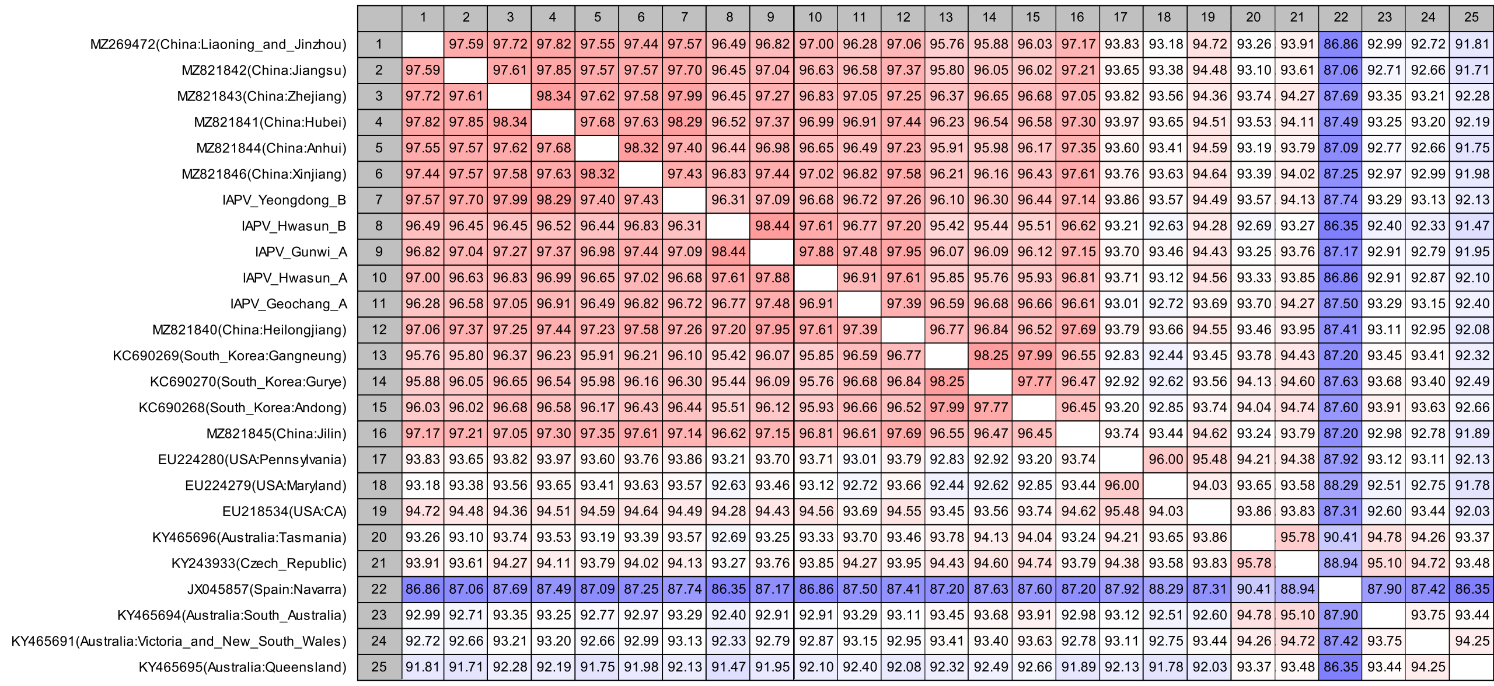


**Fig. S11** Pairwise comparison table using Israeli acute paralysis virus (IAPV) complete sequence for each country reported to NCBI GenBank database using CLC Genomics Workbench. Percent identity in 625 pairwise comparisons among 25 IAPV sequences (five sequences detected in this study and 20 sequences reported from NCBI GenBank database).


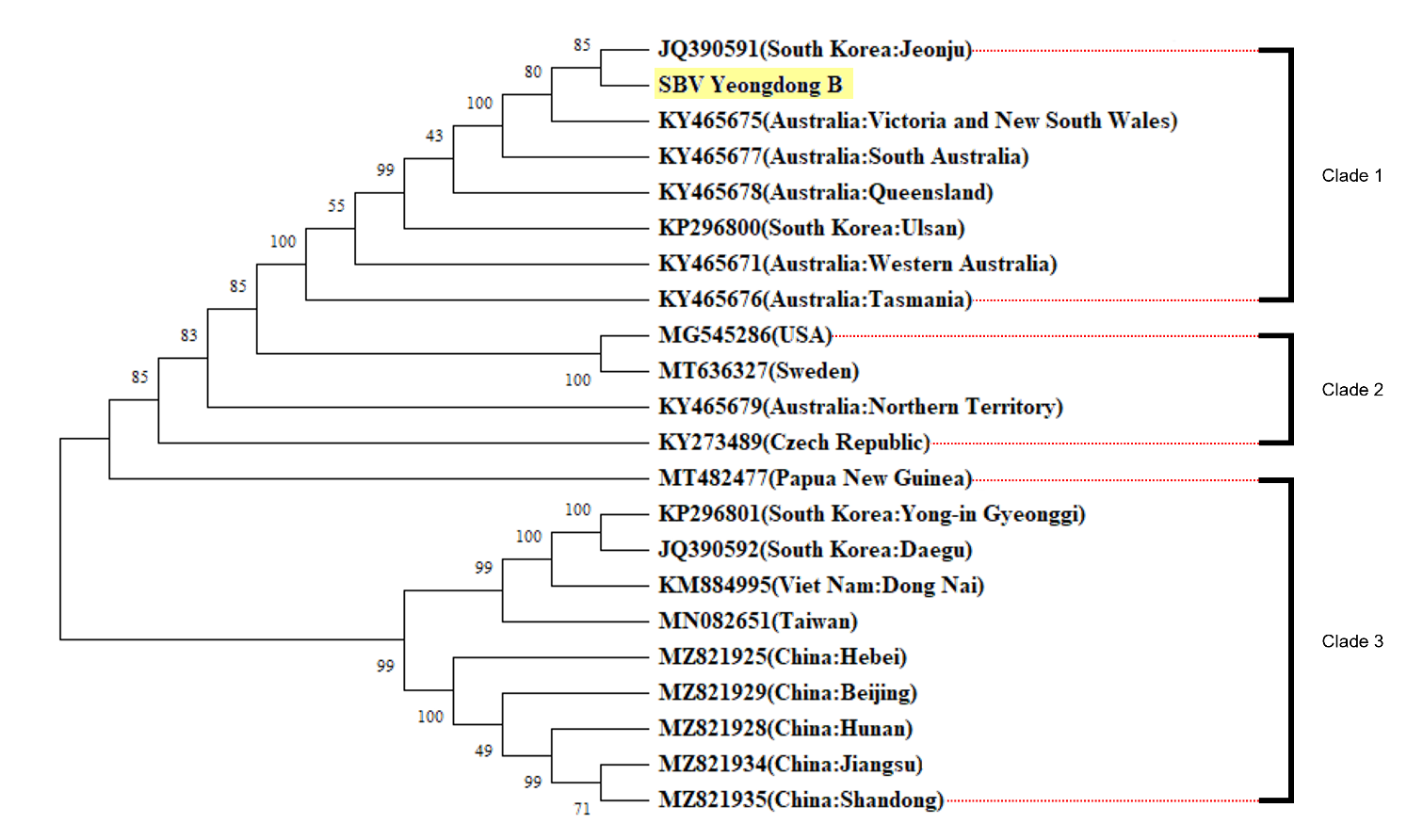


**Fig. S12** Phylogenetic tree visualizing and describing the relatedness for sacbrood virus (SBV) sequences using maximum likelihood method. The tree was developed using SBV sequences (highlighted in yellow) detected from virome analysis and other reference sequences reported in the NCBI GenBank database. The bootstrap value was from 1000 replicates and nucleotide distance was measured by Jukes-Cantor method. This tree is analyzed and visualized by MEGA11. For analysis, 21 sequences reported in the NCBI GenBank database and one sequence found in this study were used.


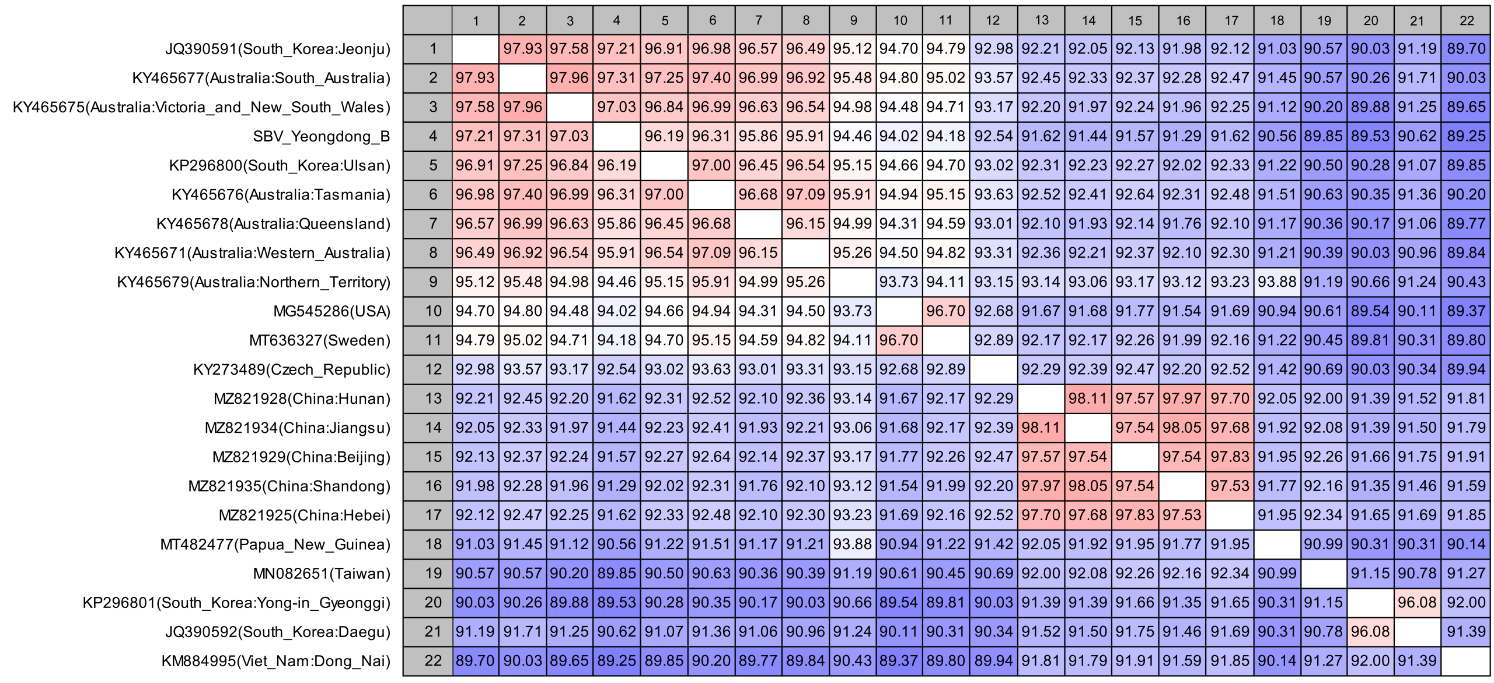


**Fig. S13** Pairwise comparison table using sacbrood virus (SBV) complete sequence for each country reported to NCBI GenBank database CLC Genomics Workbench. Percent identity in 484 pairwise comparisons among 22 SBV sequences (four sequences detected in this study and 21 sequences reported from NCBI GenBank database).


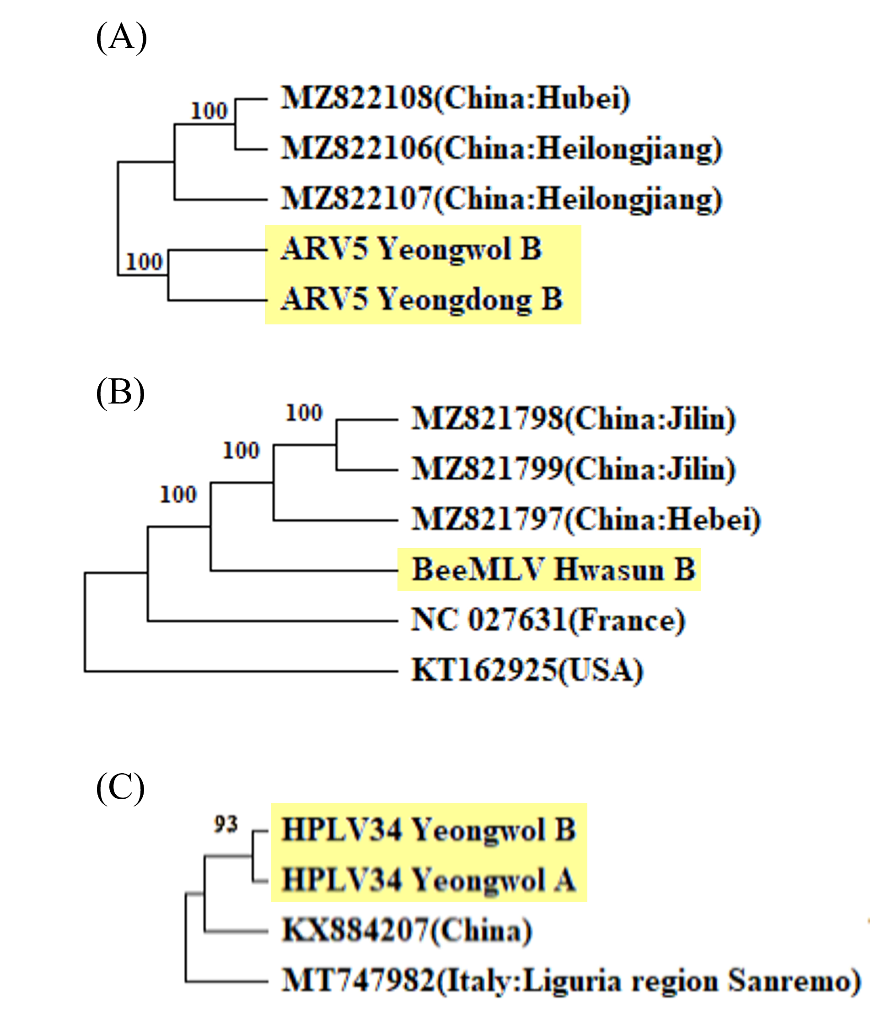


**Fig. S14** Phylogenetic tree visualizing and describing the relatedness for Apis rhabdovirus 5 (ARV5), bee macula-like virus (BeeMLV) and Hubei partiti-like virus 34 (HPLV34) sequences using maximum likelihood method. The tree was developed using ARV5, BeeMLV and HPLV34 sequences (highlighted in yellow) from detected in this study and other reference sequences reported from the NCBI GenBank database. The bootstrap value was from 1000 replicates and nucleotide distance was measured by Jukes-Cantor method. This tree is analyzed and visualized by MEGA11. (A) ARV5 (Using three sequences reported in the NCBI GenBank database and two sequences found in this study), (B) BMLV (Using five sequences reported in the NCBI GenBank database one sequences found in this study) and (C) HPLV34 (Using two sequences reported in the NCBI GenBank database and two sequences found in this study).


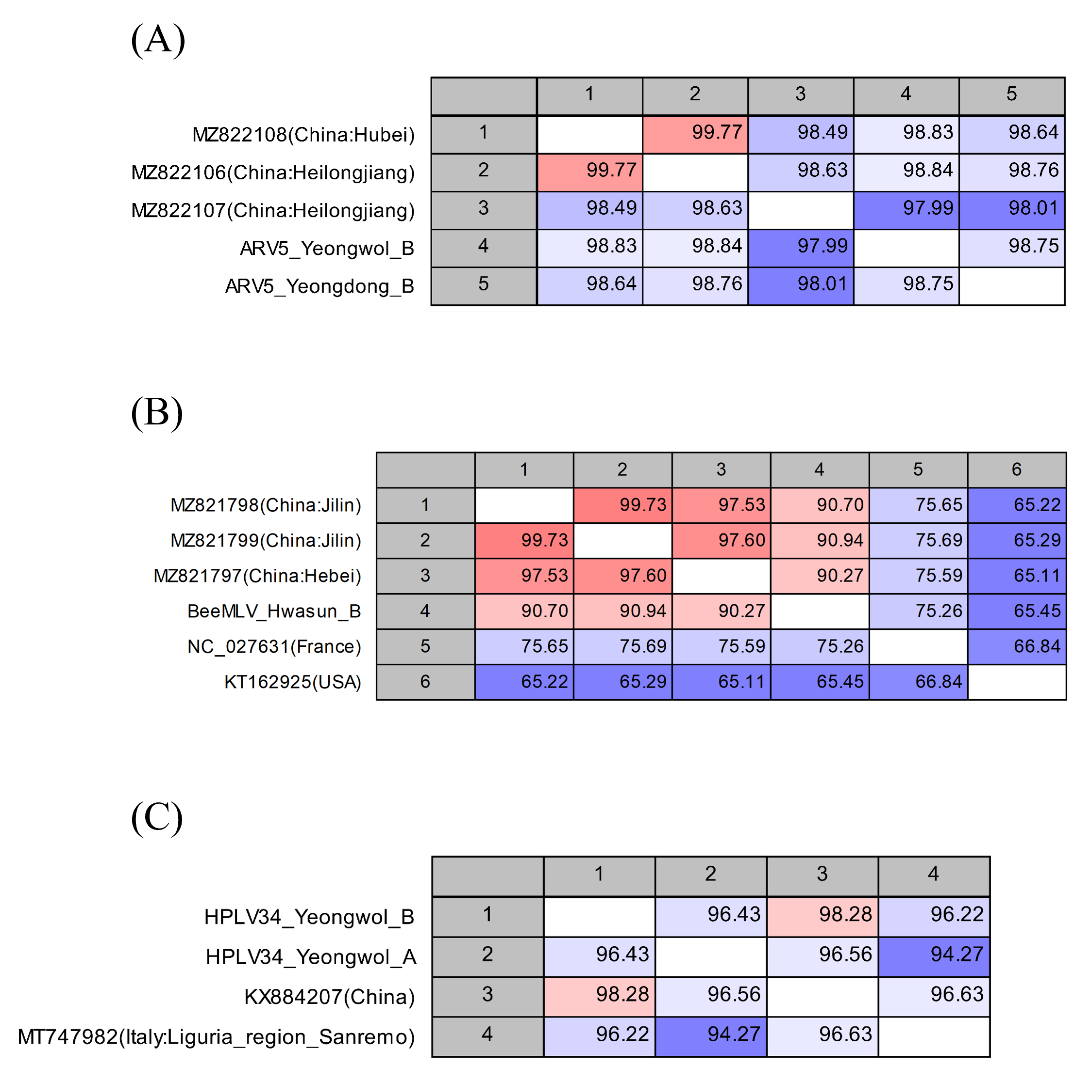


**Fig. S15** Pairwise comparison table using Apis rhabdovirus 5 (ARV5), bee macula-like virus (BeeMLV) and Hubei partiti-like virus 34 (HPLV34) complete sequence for each isolate reported to NCBI GenBank database using CLC Genomics Workbench. Percent identity in 25 pairwise comparisons among five ARV5 isolates (two isolates detected in this study and three isolates reported from NCBI GenBank database), 36 pairwise comparisons among six BeeMLV isolates (one isolate detected in this study and five isolates reported from NCBI GenBank database) and 16 pairwise comparisons among four HPLV34 isolates (one detected from virome analysis and three reported from NCBI GenBank database). (A) ARV5, (B) BMLV and (C) HPLV34.


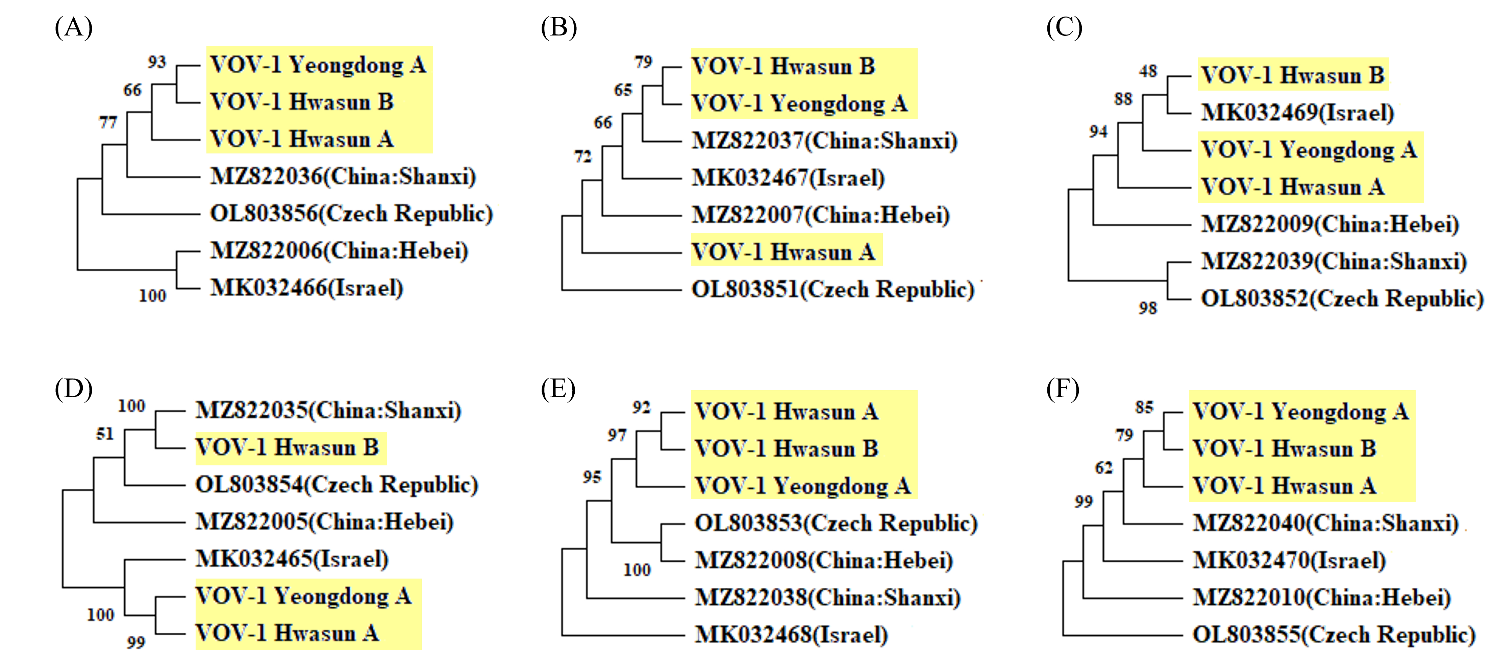


**Fig. S16** Phylogenetic tree visualizing and describing the relatedness for Varroa orthomyxovirus-1 (VOV-1) each six segment gene sequences using maximum likelihood method. The tree was developed using VOV-1 sequences (highlighted in yellow) from detected from virome analysis and other reference sequences reported from the NCBI GenBank database. The bootstrap value was from 1000 replicates and nucleotide distance was measured by Jukes-Cantor method. This tree is analyzed and visualized by MEGA11. (A) PB1 gene, (B) PB2 gene, (C) PA gene, (D) glycoprotein gene, (E) nucleoprotein gene and (F) M protein gene. For analysis, four sequences reported in the NCBI GenBank database and three sequences found in this study were used.


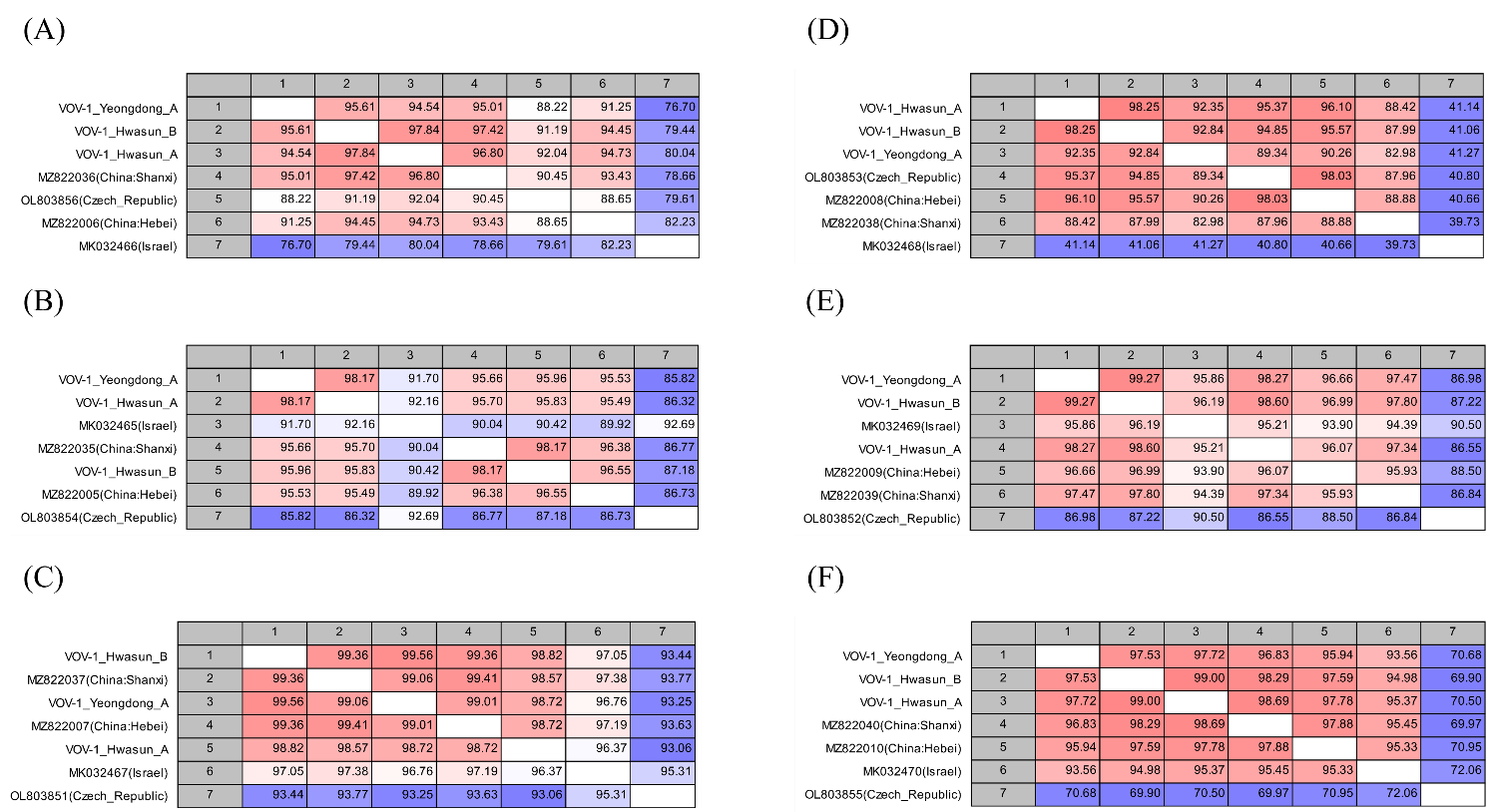


**Fig. S17** Pairwise comparison table using Varroa orthomyxovirus-1 (VOV-1) each six-segment gene complete sequence for each isolate reported to NCBI GenBank database using CLC Genomics Workbench. Percent identity in 49 pairwise comparisons among seven VOV-1 isolates (three detected from virome analysis and four reported from NCBI GenBank database). (A) PB1 gene, (B) PB2 gene, (C) PA gene, (D) glycoprotein gene, (E) nucleoprotein gene and (F) M protein gene.


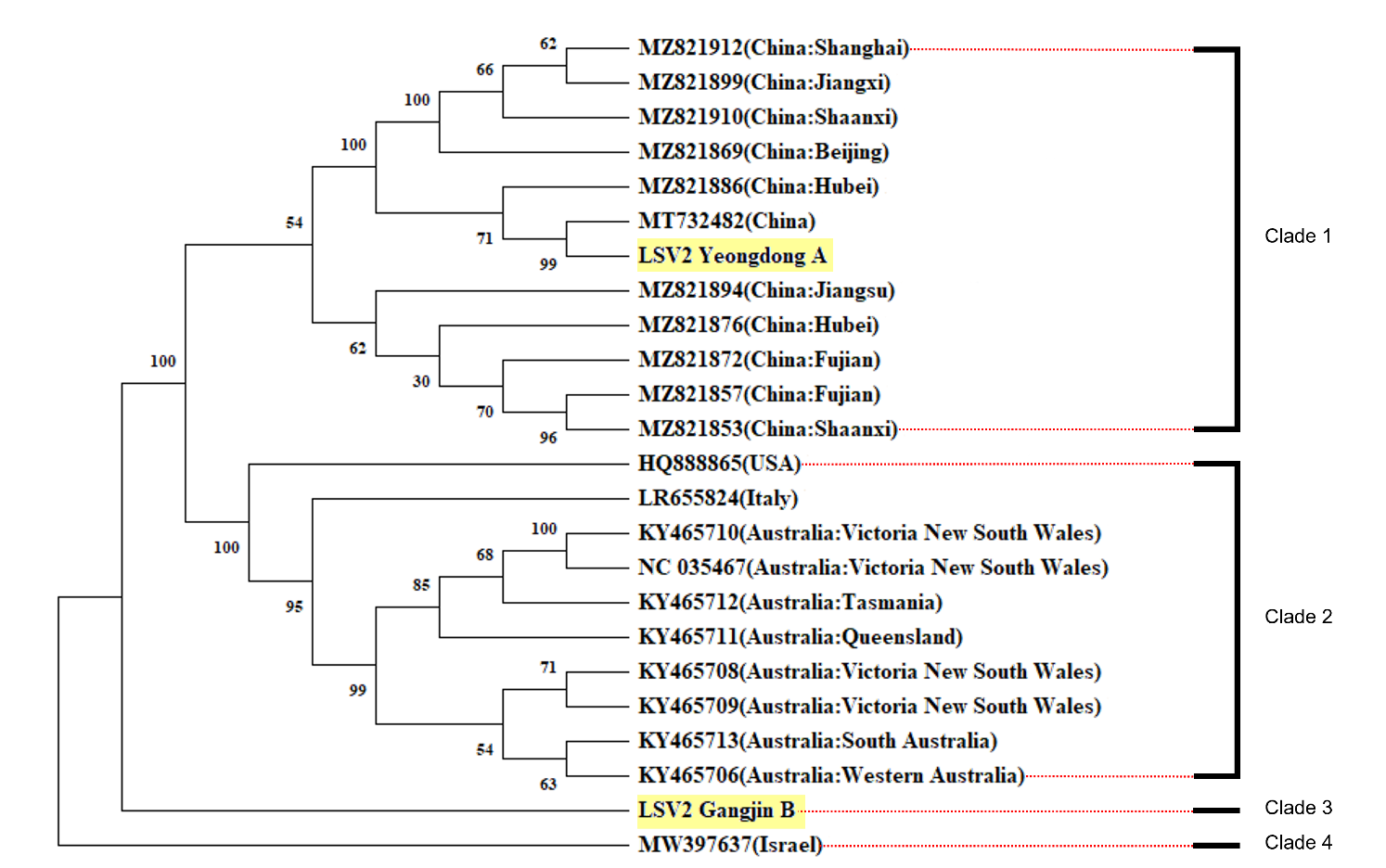


**Fig. S18** Phylogenetic tree visualizing and describing the relatedness for Lake Sinai virus 2 (LSV2) sequences using maximum likelihood method. The tree was developed using LSV2 sequences (highlighted in yellow) from detected from virome analysis and other reference sequences reported from the NCBI GenBank database. The bootstrap value was from 1000 replicates and nucleotide distance was measured by Jukes-Cantor method. This tree is analyzed and visualized by MEGA11. For analysis, 22 sequences reported in the NCBI GenBank database and two sequences found in this study were used.


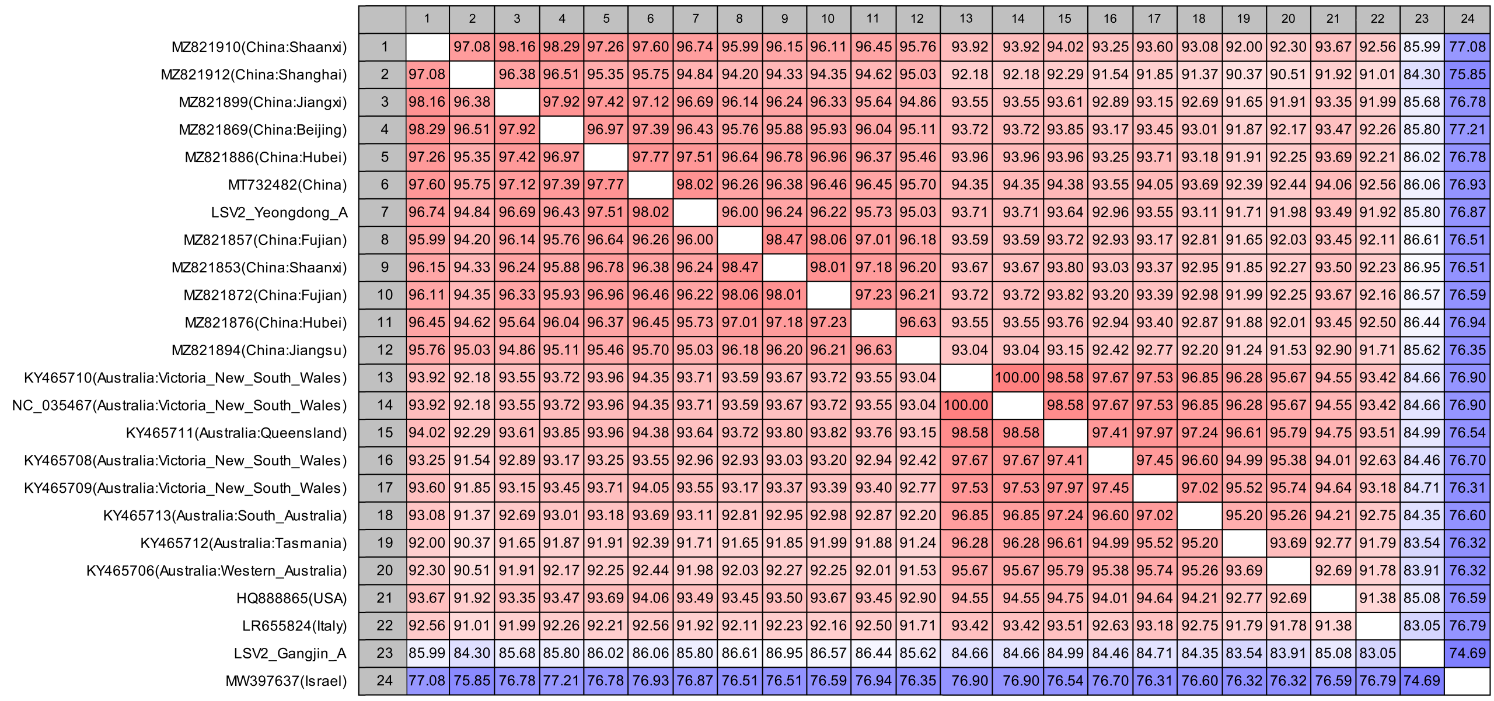


**Fig. S19** Pairwise comparison table using Lake Sinai virus 2 (LSV2) complete sequence for each isolate reported to NCBI GenBank database using CLC Genomics Workbench. Percent identity in 576 pairwise comparisons among 24 LSV2 isolates (two isolates detected in this study and 22 isolates reported from NCBI GenBank database).


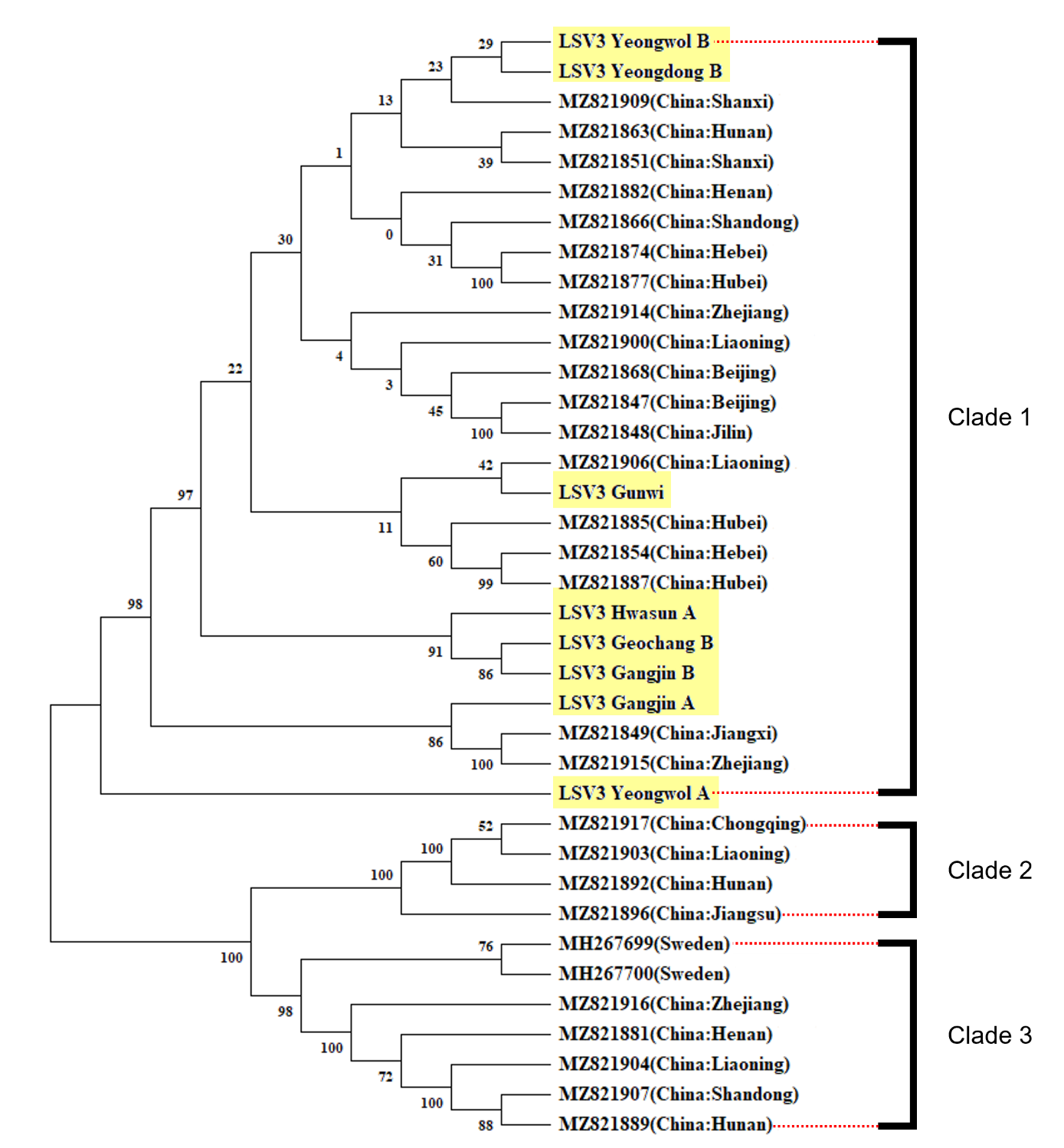


**Fig. S20** Phylogenetic tree visualizing and describing the relatedness for Lake Sinai virus 3 (LSV3) sequences using maximum likelihood method. The tree was developed using LSV3 sequences (highlighted in yellow) from detected from virome analysis and other reference sequences reported from the NCBI GenBank database. The bootstrap value was from 1000 replicates and nucleotide distance was measured by Jukes-Cantor method. This tree is analyzed and visualized by MEGA11. For analysis, 29 sequences reported in the NCBI GenBank database and eight sequences found in this study were used.


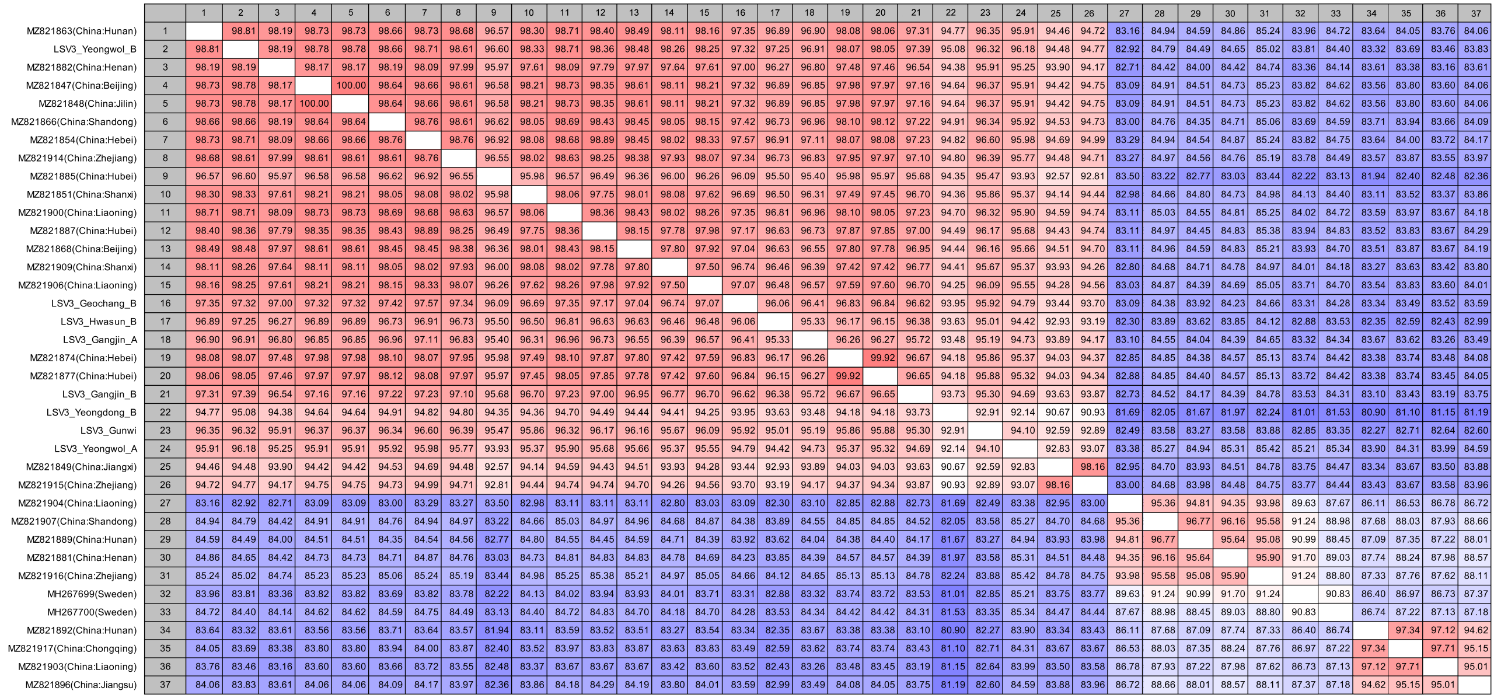


**Fig. S21** Pairwise comparison table using Lake Sinai virus 3 (LSV3) complete sequence for each isolate reported to NCBI GenBank database using CLC Genomics Workbench. Percent identity in 1,369 pairwise comparisons among 37 LSV3 isolates (eight isolates detected in this study and 29 isolates reported from NCBI GenBank database).


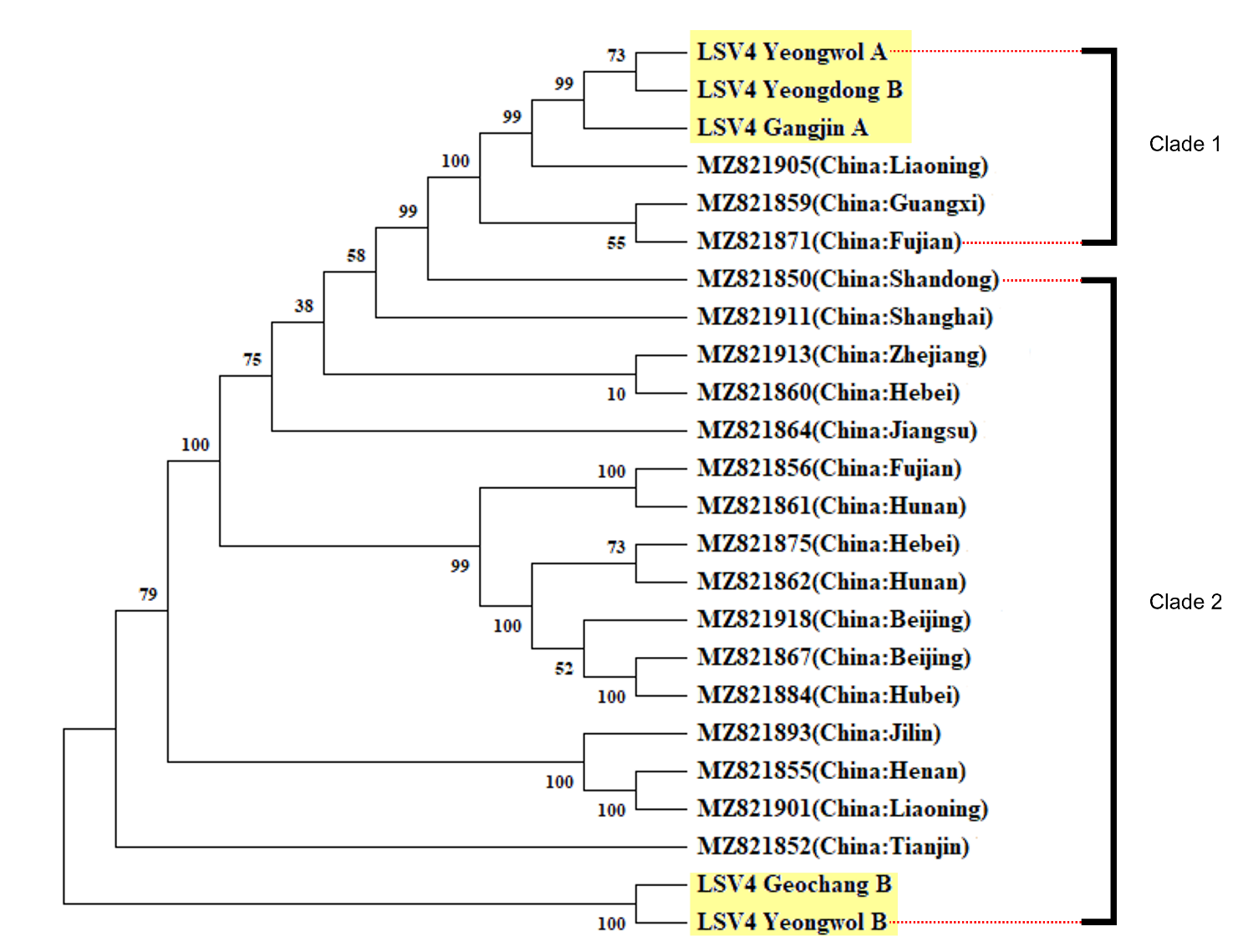


**Fig. S22** Phylogenetic tree visualizing and describing the relatedness for Lake Sinai virus 4 (LSV4) sequences using maximum likelihood method. The tree was developed using LSV4 sequences (highlighted in yellow) from detected from virome analysis and other reference sequences reported from the NCBI GenBank database. The bootstrap value was from 1000 replicates and nucleotide distance was measured by Jukes-Cantor method. This tree is analyzed and visualized by MEGA11. For analysis, 19 sequences reported in the NCBI GenBank database and five sequences found in this study were used.


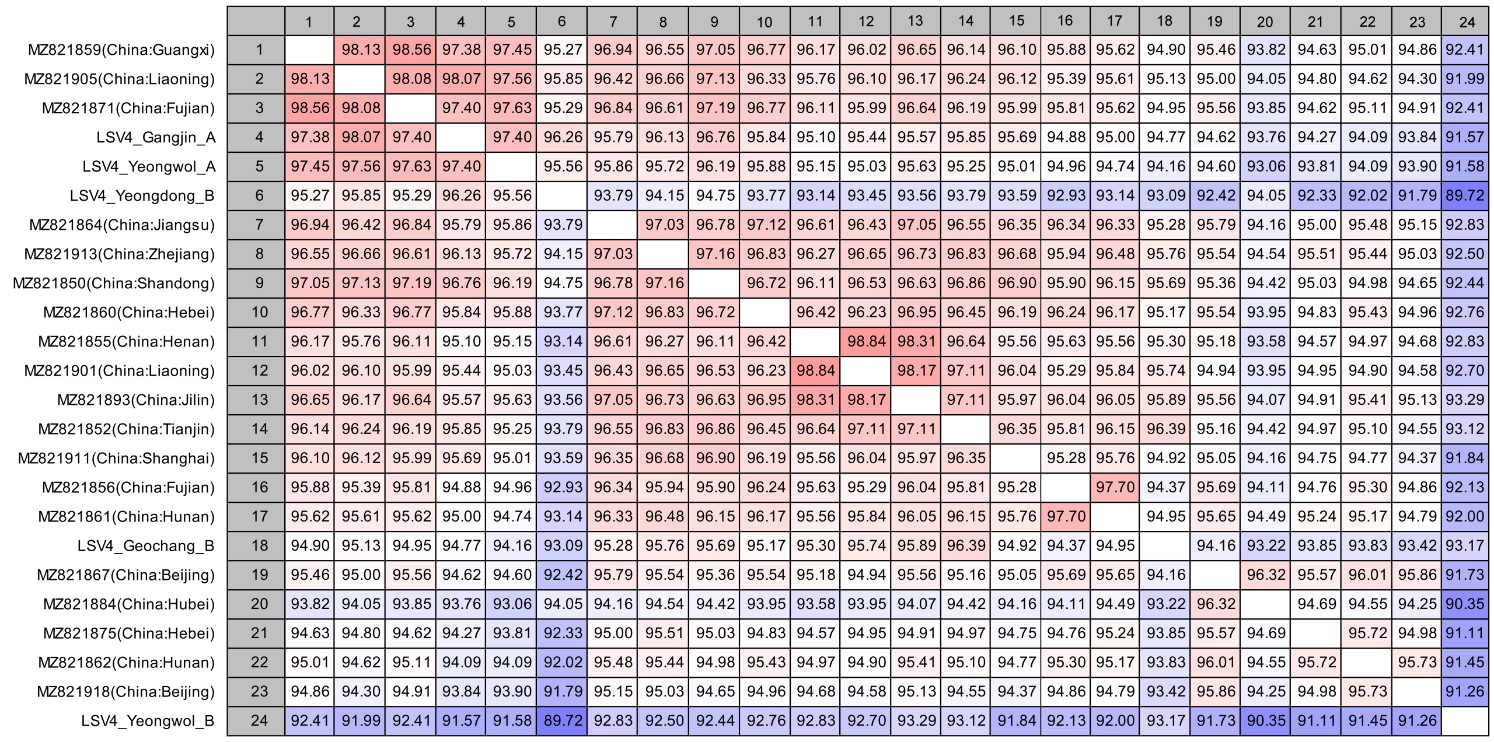


**Fig. S23** Pairwise comparison table using Lake Sinai virus 4 (LSV4) complete sequence for each isolate reported to NCBI GenBank database using CLC Genomics Workbench. Percent identity in 576 pairwise comparisons among 24 LSV4 isolates (five isolates detected in this study and 19 isolates reported from NCBI GenBank database).


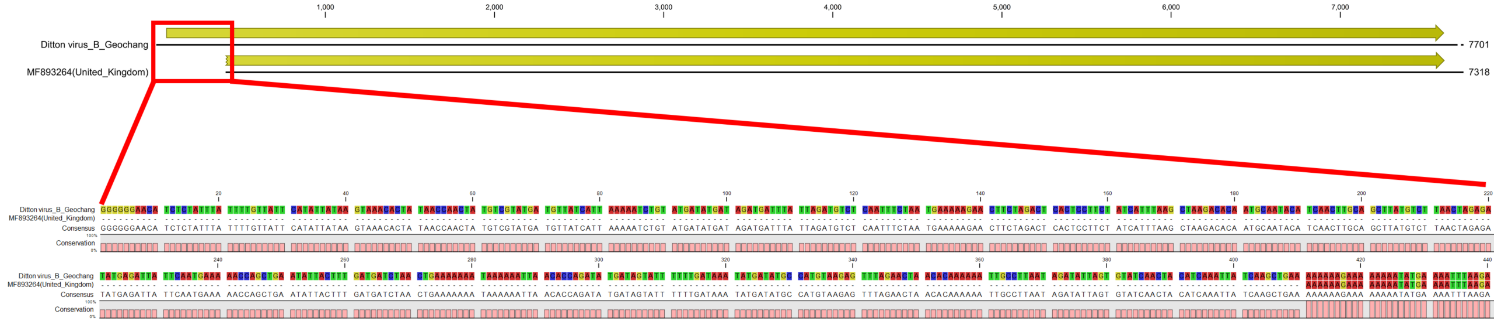


**Fig. S24** Alignment of sequence found by virome analysis with Ditton virus (DV) reported from NCBI GenBank database. Ditton virus sequences reported in NCBI GenBank and additional sequences of 410 nt length were identified.


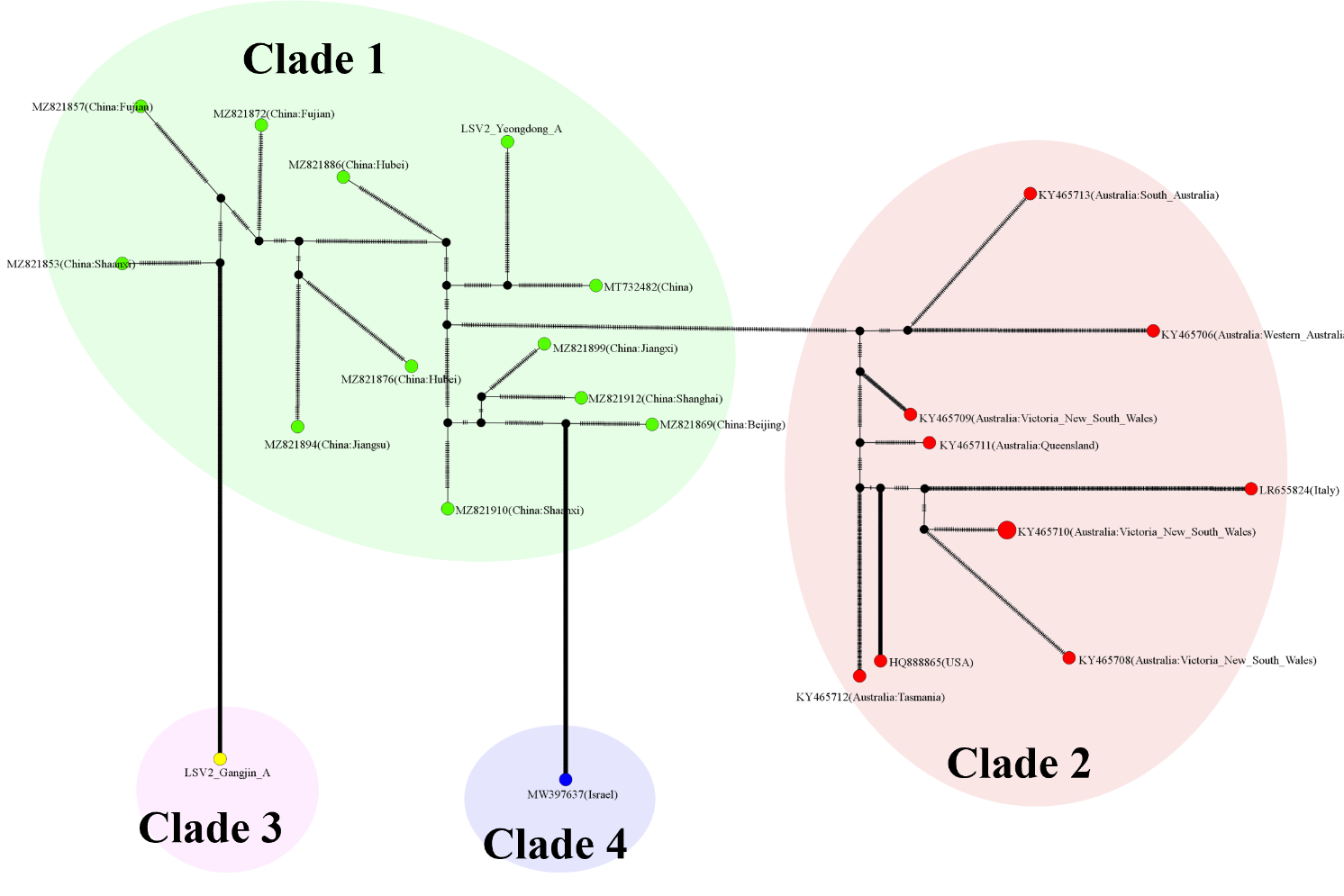


**Fig. S25** TCS haplotype network, with nodes colored by different Lake Sinai virus (LSV2) clade. A total of 23 sequences for LSV2 were used to construct the TCS network. The perpendicular dashes on the branches connecting two nodes represent the number of nucleotides difference between those nodes. This TCS network is analyzed and visualized by PopART.


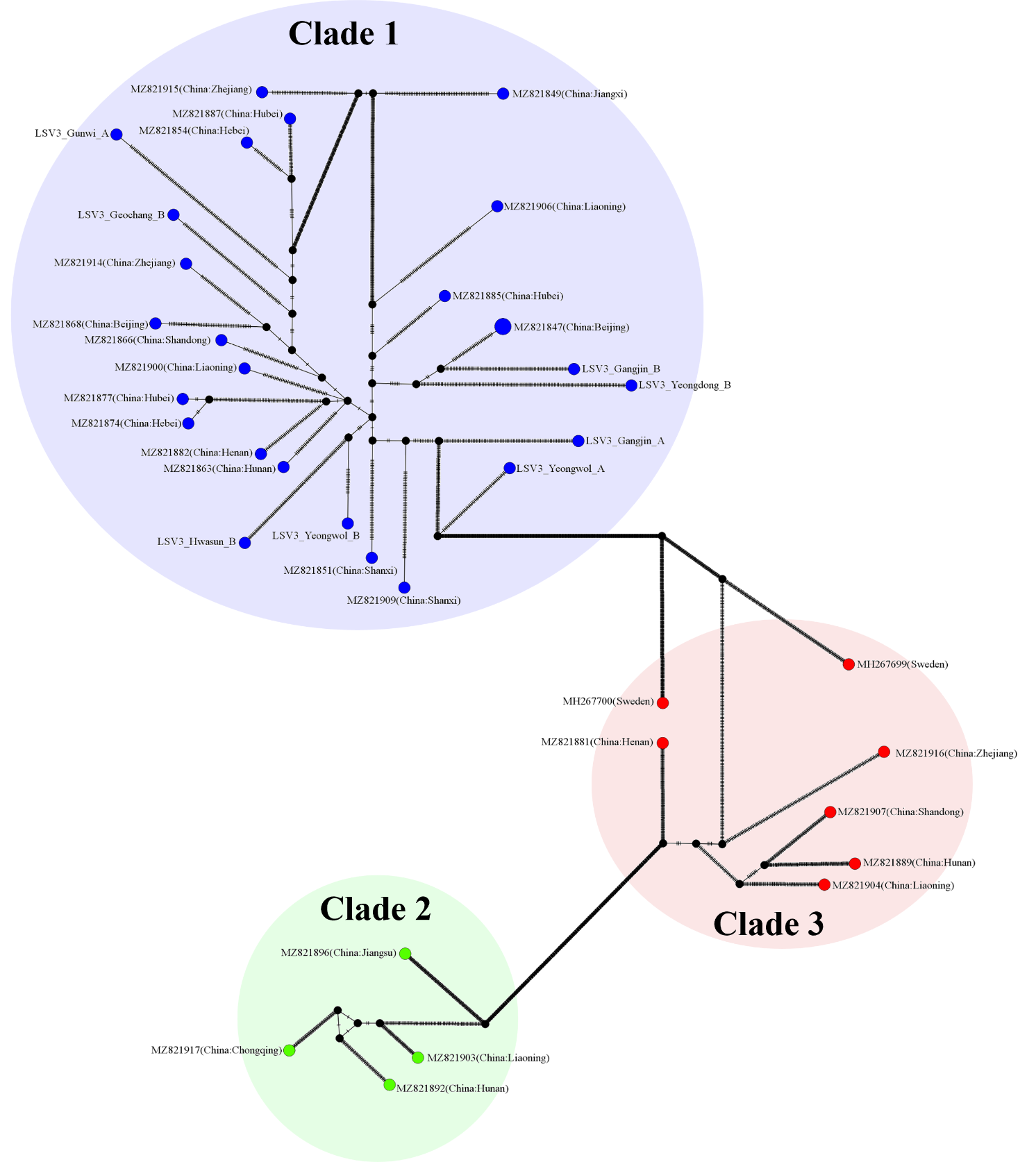


**Fig. S26** TCS haplotype network, with nodes colored by different Lake Sinai virus 3 (LSV3) clade. A total of 37 sequences for LSV3 were used to construct the TCS network. The perpendicular dashes on the branches connecting two nodes represent the number of nucleotides difference between those nodes. This TCS network is analyzed and visualized by PopART


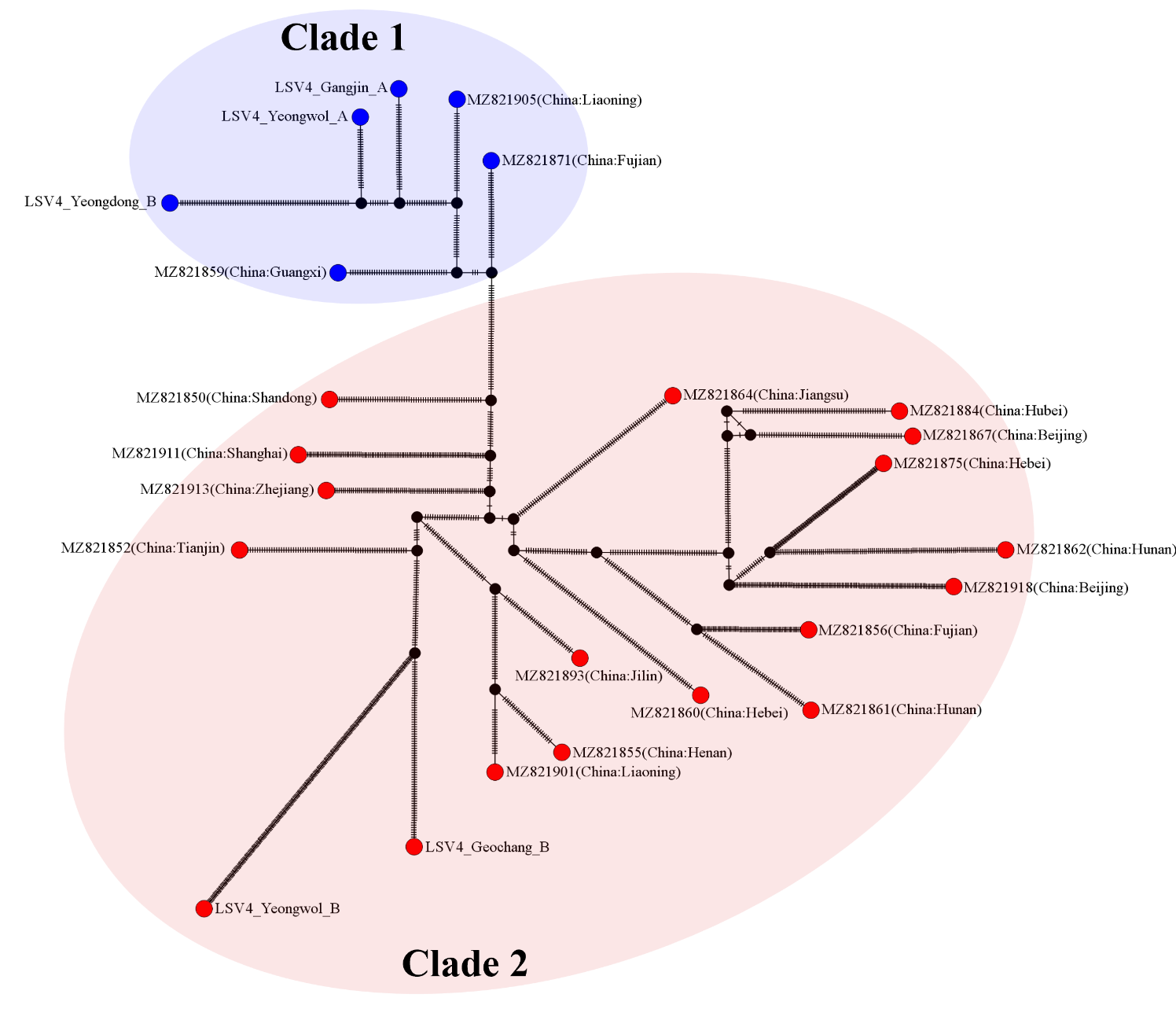


**Fig. S27** TCS haplotype network, with nodes colored by different Lake Sinai virus 4 (LSV4) clade. A total of 24 sequences for LSV4 were used to construct the TCS network. The perpendicular dashes on the branches connecting two nodes represent the number of nucleotides difference between those nodes. This TCS network is analyzed and visualized by PopART.


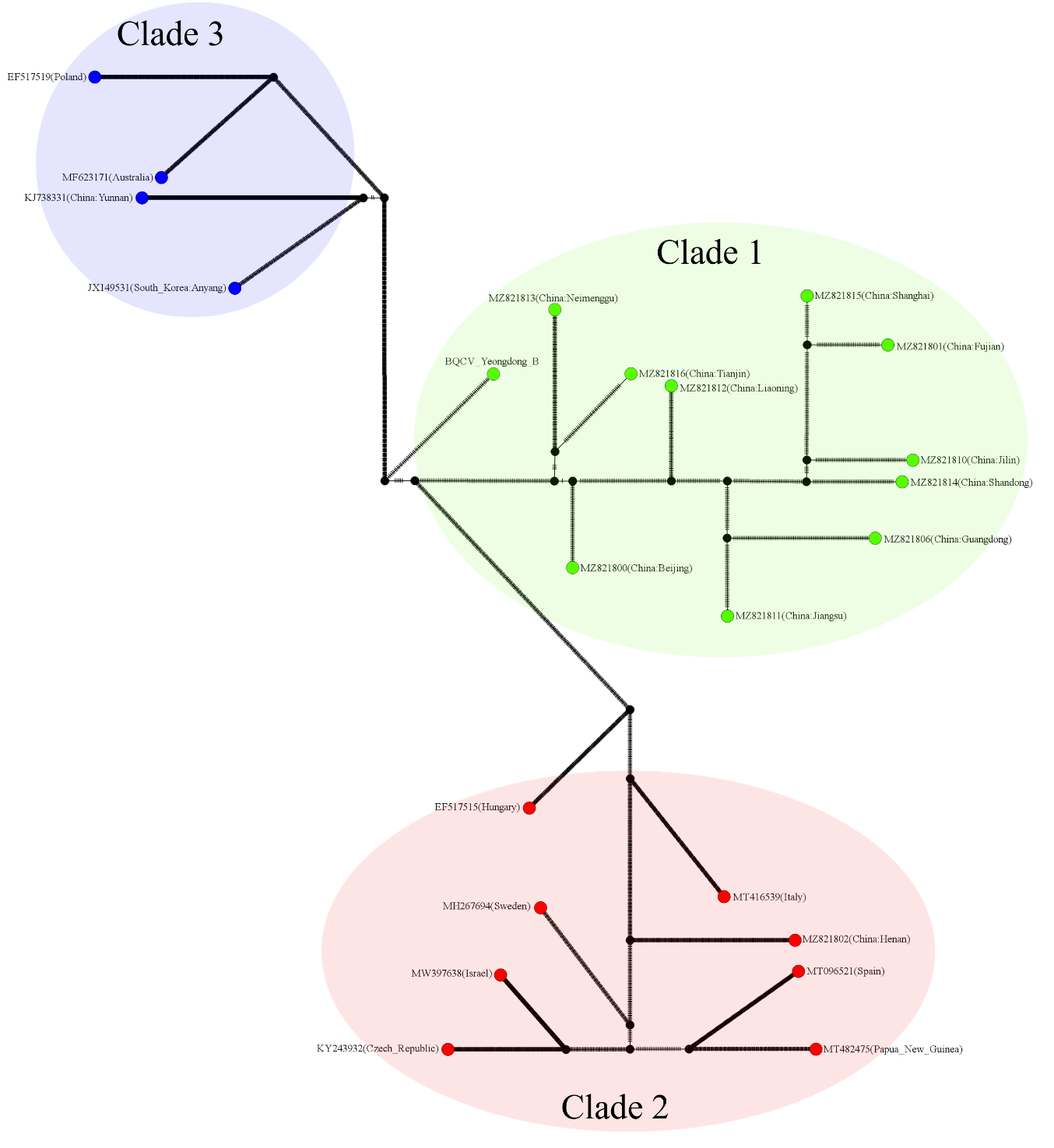


**Fig. S28** TCS haplotype network, with nodes colored by different Black queen cell virus (BQCV) clade. A total of 23 sequences for BQCV were used to construct the TCS network. The perpendicular dashes on the branches connecting two nodes represent the number of nucleotides difference between those nodes. This TCS network is analyzed and visualized by PopART.


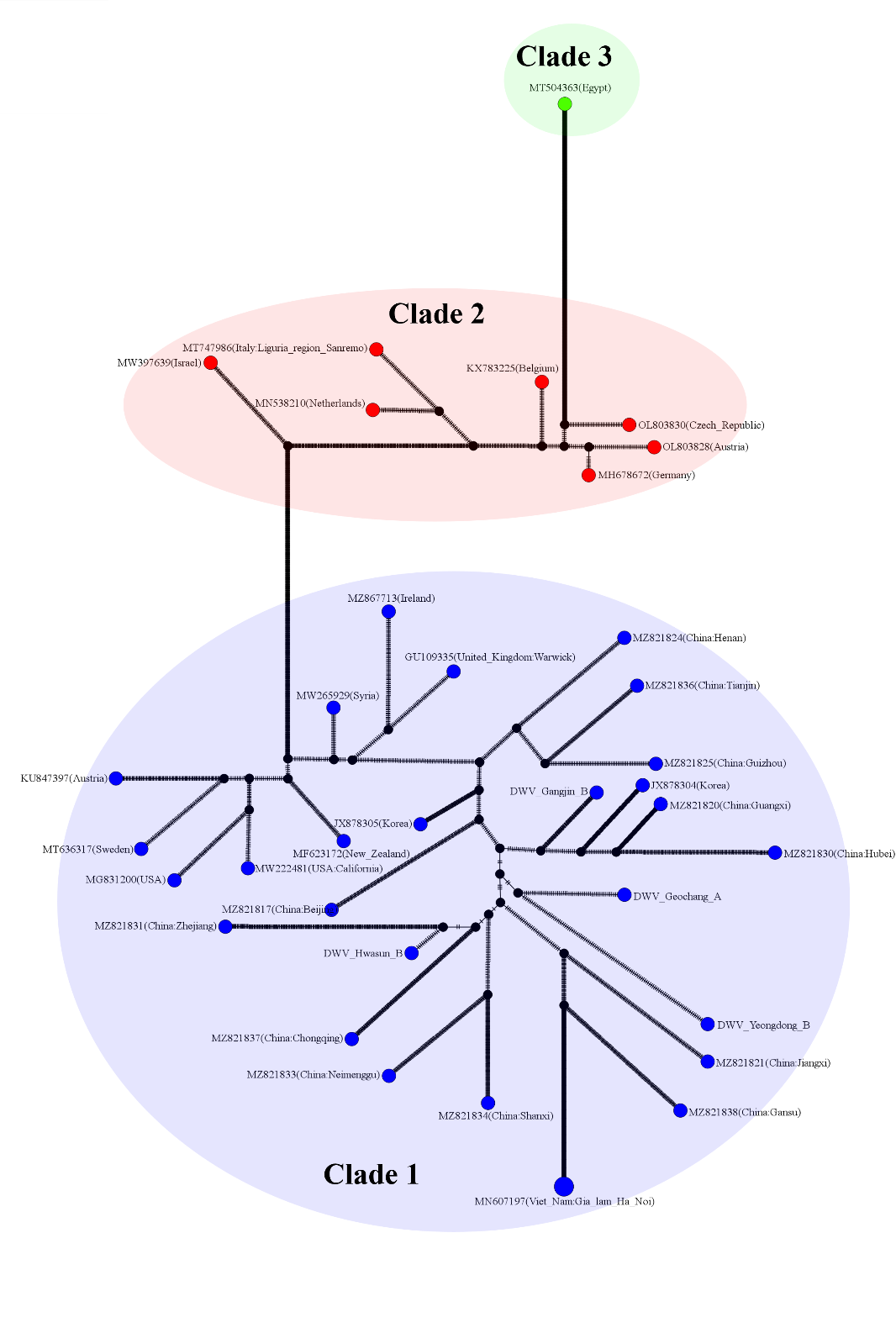


**Fig. S29** TCS haplotype network, with nodes colored by different deformed wing virus (DWV) clade. A total of 36 sequences for DWV were used to construct the TCS network. The perpendicular dashes on the branches connecting two nodes represent the number of nucleotides difference between those nodes. This TCS network is analyzed and visualized by PopART.


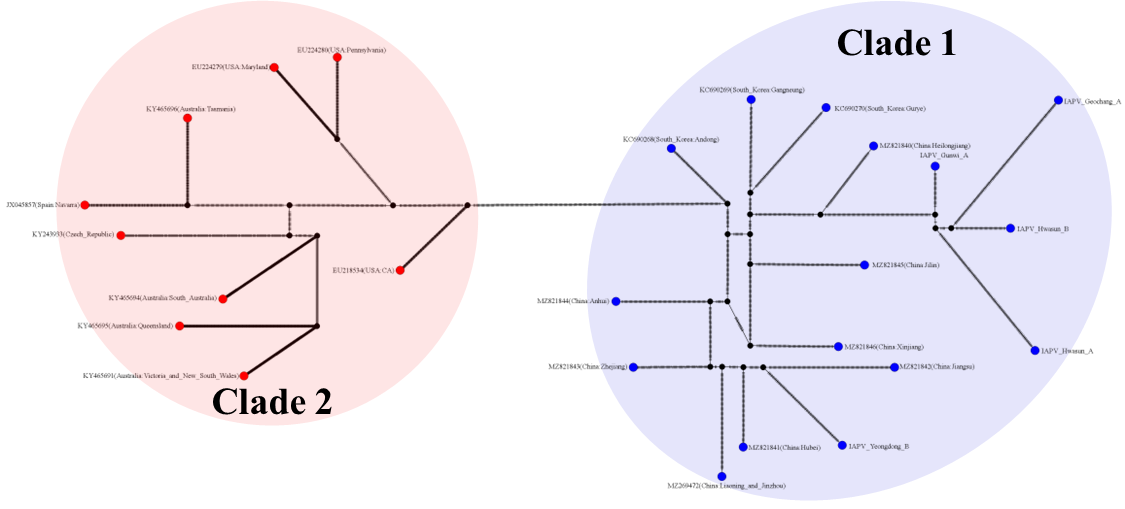


**Fig. S30** TCS haplotype network, with nodes colored by different Israeli acute paralysis virus (IAPV) clade. A total of 25 sequences for IAPV were used to construct the TCS network. The perpendicular dashes on the branches connecting two nodes represent the number of nucleotides difference between those nodes. This TCS network is analyzed and visualized by PopART.


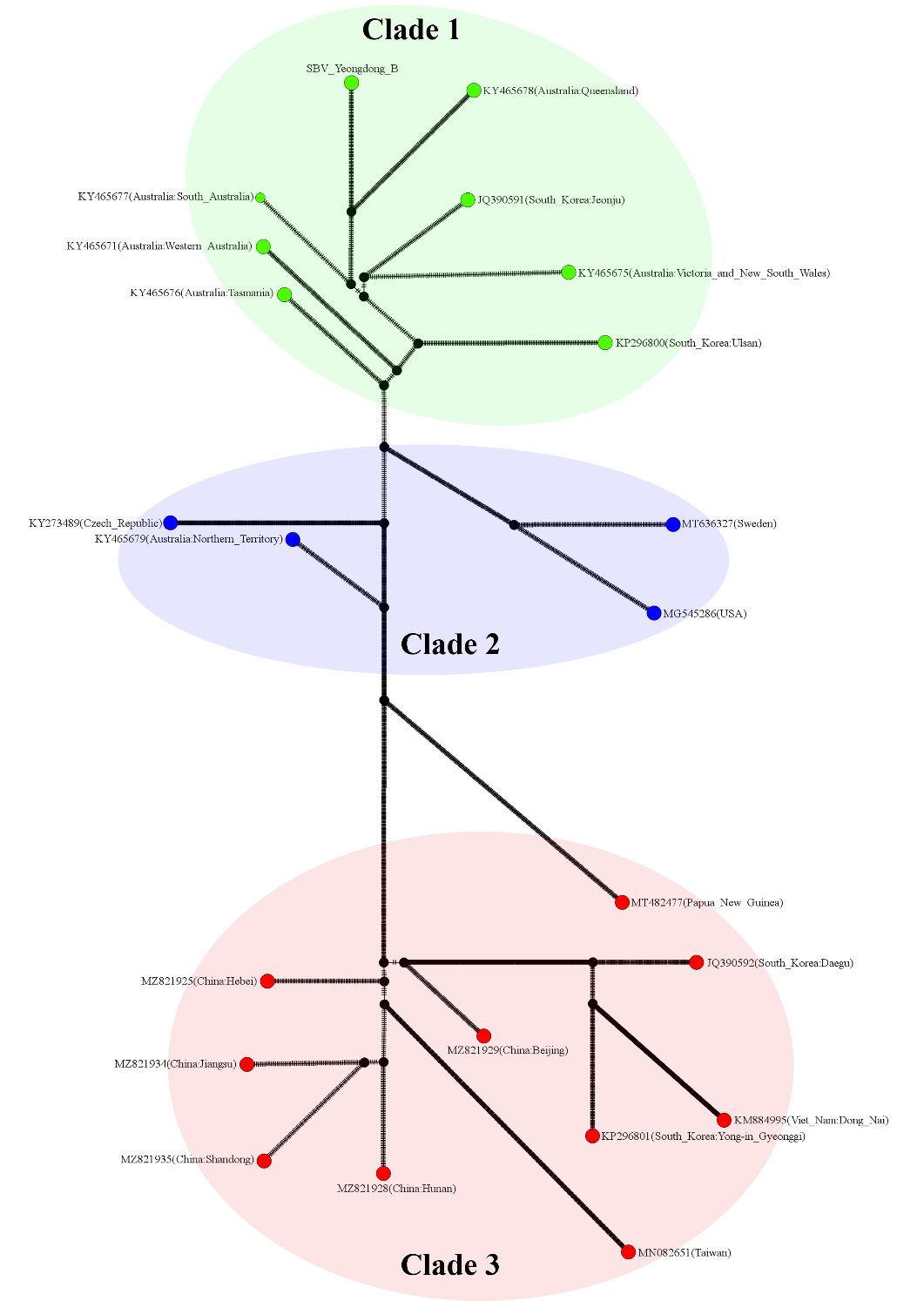


**Fig. S31** TCS haplotype network, with nodes colored by different Sacbrood virus (SBV) clade. A total of 22 sequences for SBV were used to construct the TCS network. The perpendicular dashes on the branches connecting two nodes represent the number of nucleotides difference between those nodes. This TCS network is analyzed and visualized by PopART.

**Table S1** List of viruses identified in this study

| Isolate | | Virus | Length (nt) | High identity (%; Acc) | Accession |
| --- | --- | --- | --- | --- | --- |
| Group "A" | Yeongdong | VOV-1 PA | 2,030 | 99.06 (MZ822037) | OP972904 |
|  |  | VOV-1 PB1 | 2,343 | 95.01 (MZ822036) | OP972907 |
|  |  | VOV-1 PB2 | 2,349 | 95.66 (MZ822035) | OP972910 |
|  |  | VOV-1 glycoprotein | 1,745 | 90.26 (MZ822008) | OP972913 |
|  |  | VOV-1 nucleoprotein | 1,498 | 97.47 (MZ822039) | OP972923 |
|  |  | VOV-1 M protein | 1,009 | 96.83 (MZ822040) | OP972900 |
|  |  | LSV2 | 5,897 | 98.02 (MT732482) | OP972884 |
|  |  | AmCV RNA1 | 5,899 | 76.28 (MH899120) | OP972917 |
|  |  | AmCV RNA2 | 3,442 | 75.86 (MK231039) | OP972919 |
|  | Gangjin | LSV2 | 5,952 | 86.95 (MZ821853) | OP972922 |
|  |  | LSV3 | 6,090 | 97.39 (MZ821847) | OP972885 |
|  |  | LSV4 | 6,076 | 98.07 (MZ821905) | OP972893 |
|  | Hwasun | VOV-1 PA | 2,032 | 98.72 (MZ822007) | OP972902 |
|  |  | VOV-1 PB1 | 2,250 | 96.80 (MZ822036) | OP972905 |
|  |  | VOV-1 PB2 | 2,346 | 95.70 (MZ822035) | OP972908 |
|  |  | VOV-1 glycoprotein | 1,641 | 96.10 (MZ822008) | OP972911 |
|  |  | VOV-1 nucleoprotein | 1,502 | 97.34 (MZ822039) | OP972914 |
|  |  | VOV-1 M protein | 993 | 98.69 (MZ822040) | OP972899 |
|  |  | IAPV | 9,602 | 97.61 (MZ821840) | OP972881 |
|  | Geochang | DWV | 10,104 | 97.04 (JX878305) | OP972874 |
|  |  | IAPV | 9,502 | 97.39 (MZ821840) | OP972880 |
|  | Yeongwol | HPLV34 | 1,484 | 96.56 (KX884207) | OP972878 |
|  |  | LSV3 | 5,998 | 96.18 (MZ821847) | OP972891 |
|  |  | LSV4 | 5,992 | 97.63 (MZ821871) | OP972896 |
|  | Gunwi | IAPV | 9,566 | 97.95 (MZ821840) | OP972916 |
|  |  | LSV3 | 6,042 | 96.37 (MZ821847 and MZ821848) | OP972887 |
| Group "B" | Yeongdong | BQCV | 8,457 | 96.87 (MZ821814) | OP972872 |
|  |  | DWV | 10,157 | 96.54 (JX878305) | OP972876 |
|  |  | IAPV | 9,550 | 98.29 (MZ821841) | OP972883 |
|  |  | SBV | 8,842 | 97.31 (KY465677) | OP972898 |
|  |  | ARV5 | 13,389 | 98.76 (MZ822106) | OP972869 |
|  |  | LSV3 | 6,153 | 94.91 (MZ821866) | OP972890 |
|  |  | LSV4 | 6,147 | 95.85 (MZ821905) | OP972895 |
|  |  | AmPLV1 | 1,325 | 96.48 (JF732915) | OP972921 |
|  |  | AmCV RNA1 | 5,921 | 76.50 (MH899120) | OP972918 |
|  |  | AmCV RNA2 | 3,608 | 76.01 (MK231039) | OP972920 |
|  | Gangjin | DWV | 10,021 | 94.35 (JX878305) | OP972873 |
|  |  | LSV3 | 6,061 | 97.31 (MZ821863) | OP972886 |
|  | Hwasun | DWV | 10,159 | 97.32 (MZ821833) | OP972875 |
|  |  | IAPV | 9,633 | 97.20 (MZ821840) | OP972882 |
|  |  | VOV-1 PA | 2,028 | 99.36 (MZ822037 and MZ822007) | OP972903 |
|  |  | VOV-1 PB1 | 2,271 | 97.42 (MZ822036) | OP972906 |
|  |  | VOV-1 PB2 | 2,348 | 98.17 (MZ822035) | OP972909 |
|  |  | VOV-1 glycoprotein | 1,649 | 95.57 (MZ822008) | OP972912 |
|  |  | VOV-1 nucleoprotein | 1,495 | 97.80 (MZ822039) | OP972915 |
|  |  | VOV-1 M protein | 996 | 98.29 (MZ822040) | OP972901 |
|  |  | BeeMLV | 6,280 | 90.70 (MZ821798) | OP972871 |
|  |  | LSV3 | 6,077 | 96.91 (MZ821854) | OP972889 |
|  | Geochang | LSV3 | 6,080 | 97.57 (MZ821854) | OP972888 |
|  |  | LSV4 | 6,039 | 96.39 (MZ821852) | OP972894 |
|  |  | DV | 7,701 | 98.89 (MF893264) | OP972877 |
|  | Yeongwol | ARV5 | 13,405 | 98.84 (MZ822106) | OP972870 |
|  |  | HPLV34 | 1,449 | 98.28 (KX884207) | OP972879 |
|  |  | LSV3 | 6,043 | 98.81 (MZ821863) | OP972892 |
|  |  | LSV4 | 5,986 | 92.83 (MZ821864 and MZ821855) | OP972897 |

**Table S2** BLAST results for novel viral contigs

| Sequence information | BLAST type | Description | Max  score | Total  score | Query  cover | E  value | Per.  Ident | Acc.  Len | Accession |
| --- | --- | --- | --- | --- | --- | --- | --- | --- | --- |
| Yeongdong of group B | BLASTn | Uncultured virus | 797 | 797 | 36% | 0.0 | 96.48% | 483 | JF732915.1 |
|  | BLASTn | Vespa velutina partiti-like virus 2 | 595 | 595 | 98% | 1e-164 | 70.41% | 1613 | MN565048.1 |
| Yeongdong of group B RdRp region | tBLASTx | Vespa velutina partiti-like virus 2 | 791 | 2771 | 99% | 0.0 | 71.99% | 1613 | MN565048.1 |

**Table S3** BLASTx results for plant virus-associated contigs

| **Sequence information** | **Description** | **Max**  **score** | **Total**  **score** | **Query**  **cover** | **E**  **value** | **Per.**  **Ident** | **Acc.**  **Len** | **Accession** |
| --- | --- | --- | --- | --- | --- | --- | --- | --- |
| Yeongdong of group A RNA1 | Arabidopsis latent virus-1 | 3103 | 3103 | 99% | 0.0 | 76.28% | 5953 | MH899120.1 |
|  | Zymoseptoria comovirus | 3086 | 3086 | 99% | 0.0 | 76.23% | 5974 | MK231051.1 |
|  | Radish mosaic virus | 106 | 106 | 3% | 5e-17 | 75.93% | 6048 | EU450837.1 |
|  | Fabavirus safflower/YL-2010/CHN | 71.3 | 71.3 | 0% | 2e-06 | 93.75% | 2312 | GU380344.1 |
| Yeongdong of group A RNA2 | Zymoseptoria comovirus A | 1356 | 1356 | 78% | 0.0 | 75.86% | 3573 | MK231039.1 |
|  | Arabidopsis latent virus-1 | 1336 | 1336 | 78% | 0.0 | 75.71% | 3600 | MH899121.1 |
|  | Arabidopsis latent virus-1 | 71.3 | 71.3 | 1% | 1e-06 | 90.74% | 5953 | MH899120.1 |
|  | Zymoseptoria comovirus | 71.3 | 71.3 | 1% | 1e-06 | 90.74% | 5974 | MK231051.1 |
| Yeongdong of group B RNA1 | Arabidopsis latent virus-1 | 3158 | 3158 | 99% | 0.0 | 76.50% | 5953 | MH899120.1 |
|  | Zymoseptoria comovirus | 1318 | 1318 | 99% | 0.0 | 76.46% | 5974 | MK231051.1 |
|  | Radish mosaic virus | 111 | 111 | 3% | 1e-18 | 76.39% | 6048 | EU450837.1 |
|  | Fabavirus safflower/YL-2010/CHN | 71.3 | 71.3 | 0% | 2e-06 | 93.75% | 2312 | GU380344.1 |
| Yeongdong of group B RNA2 | Zymoseptoria comovirus A | 1378 | 1378 | 74% | 0.0 | 76.01% | 3573 | MK231039.1 |
|  | Arabidopsis latent virus-1 | 1358 | 1358 | 74% | 0.0 | 75.85% | 3600 | MH899121.1 |
|  | Arabidopsis latent virus-1 | 145 | 145 | 3% | 6e-29 | 87.90% | 5953 | MH899120.1 |
|  | Zymoseptoria comovirus | 145 | 145 | 3% | 6e-29 | 87.90% | 5974 | MK231051.1 |
